# *Lactuca racemosa* Willd., Source of Antioxidants with Diverse Chemical Structures

**DOI:** 10.3390/molecules29245975

**Published:** 2024-12-18

**Authors:** Klaudia Michalska, Danuta Jantas, Janusz Malarz, Klaudia Jakubowska, Wojciech Paul, Anna Stojakowska

**Affiliations:** 1 Maj Institute of Pharmacology, Polish Academy of Sciences, Smętna Street 12, 31-343 Kraków, Poland; jantas@if-pan.krakow.pl (D.J.); malarzj@if-pan.krakow.pl (J.M.);; 2 W. Szafer Institute of Botany, Polish Academy of Sciences, Lubicz 46, 31-512 Kraków, Poland; w.paul@botany.pl

**Keywords:** austricin, caffeoylquinic acids, caffeoyltartaric acids, *Cicerbita*, Cichorieae, deacetylmatricarin, hydrogen peroxide, neuroprotection, 6-OHDA, SH-SY5Y cells

## Abstract

Ethanolic extracts from the roots and aerial parts of the hitherto chemically uninvestigated lettuce species *Lactuca racemosa* Willd. (Cichorieae, Asteraceae) were chromatographically separated to obtain eight sesquiterpenoids, two apocarotenoids (loliolide and (6*S*,9*S*) roseoside), and three phenolic glucosides (apigenin 7-*O*-glucoside, eugenyl-4-*O*-*β*-glucopyranoside, and 5-methoxyeugenyl-4-*O*-*β*-glucopyranoside). Four of the isolated sesquiterpene lactones (8-*α*-angeloyloxyleucodin, matricarin, 15-deoxylactucin, and deacetylmatricarin 8-*β*-glucopyranoside) have not previously been found either in *Lactuca* spp. or in *Cicerbita* spp. In addition, HPLC-PAD chromatographic methods were used to estimate the deacetylmatricarin derivatives, luteolin 7-*O*-glucoside, and caffeic acid derivatives contents in the analyzed plant material. The aerial parts contained c. 3.0% dry weight of chicoric acid and equal amounts (0.4%) of caftaric acid and luteolin 7-*O*-glucoside. The roots contained fewer phenolic metabolites but were rich in deacetylmatricarin glucoside (c. 1.3%). The aglycone of the most abundant sesquiterpene lactone was evaluated with respect to its neuroprotective effect in H_2_O_2_- and 6-OHDA-treated human neuroblastoma SH-SY5Y cells. This compound, at concentrations of 10 and 50 μM, provided partial protection of undifferentiated cells, and at a concentration of 50 μM, it provided partial protection of retinoic acid-differentiated cells from H_2_O_2_-induced damage. In a model of 6-OHDA-evoked cytotoxicity, the sesquiterpenoid was less effective. Our findings may support the inclusion of this plant into the human diet.

## 1. Introduction

The current taxonomic classification [1,2,3] places *Lactuca racemosa* Willd. (Cichorieae, synonym: Lactuceae; Asteraceae) in the genus *Lactuca* L., together with c. 150 other taxa. Some sources have placed (or still place) the taxon within the close genus *Cicerbita*, as *Cicerbita racemosa* (Willd.) Beauverd [4,5]. Based on plastid DNA and nuclear ribosomal internal transcribed spacer (nrITS) datasets, it has been proven that *L. racemosa* is closely related to *L. macrophylla* A. Gray and *L. adenophora* Boiss. and Kotschy, with which species it forms a separate and exclusive (ITS) or almost exclusive (plastid DNA) clade within the *Lactuca* genus [6,7]. *L. racemosa* is a perennial herb, native to the Caucasus and Turkey [6]. Though numerous species of the tribe Cichorieae are known as wild food plants or seasonal vegetables (e.g., *Cicerbita* spp., *Cichorium* spp., *Crepis* spp., *Lactuca* spp., *Scorzonera* spp., *Taraxacum* spp., and *Tragopogon* spp.), data regarding the use of *L. racemosa* are scarce. The aerial parts of the species may be used regionally to prepare salads, and in the Rize region of Turkey are utilized as a forage [8,9]. Also, little is known about the metabolites synthesized by the plant. Kaltalioğlu and coworkers [9], using high-performance liquid chromatography (HPLC) with photodiode array (PDA) detection and a dozen of standards, detected protocatechuic acid, *p*-OH-benzoic acid, chlorogenic acid (5-caffeoylquinic acid, 5-CQA), caffeic acid, p-coumaric acid, and quercetin in a methanol extract from the aerial parts of *L. racemosa*. However, the main signals observed in the chromatograms remained unidentified. The extract demonstrated moderate antioxidant activity in comparison with the concomitantly analyzed *Hypericum scabrum* L. and *Laser trilobum* L. extracts. The genetically related species *L. macrophylla* and *L. adenophora* (Turkish endemite) have not been chemically investigated so far [10].

The *Lactuca* genus comprises numerous cultivars of the popular leaf vegetable *L. sativa* L. and several species which are the gene pool for the cultivated plant. The best-known specialized metabolites produced by the *Lactuca* plants, which are believed to carry some health benefits [11], are flavonoids (mainly the derivatives of quercetin and luteolin), hydroxycinnamic acids (including chicoric acid and 5-CQA), and sesquiterpene lactones of different skeletal types [12,13].

As a part of a study on the specialized metabolites of the genus *Lactuca* [12,13,14,15,16], a phytochemical analysis of the roots and aerial parts of *L. racemosa* has been performed, and the protective activity of deacetylmatricarin (austricin; major sesquiterpene lactone found in the plant) against hydrogen peroxide (H_2_O_2_)- or 6-hydroxydopamine (6-OHDA)-evoked cell damage in both undifferentiated (UN-SH-SY5Y) and differentiated (RA-SH-SY5Y) human neuroblastoma SH-SY5Y cells has been investigated. The aim of this study is to determine the chemical compositions of the extracts from the yet unexplored plant in a search for biologically active terpenoid and phenolic metabolites, to assess the value of the plant as a component of the diet, and to provide further insight into the chemophenetic relationships within the genus *Lactuca*. 

## 2. Results

### 2.1. Isolation of Specialized Metabolites from L. racemosa Extracts

**Figure 1:**
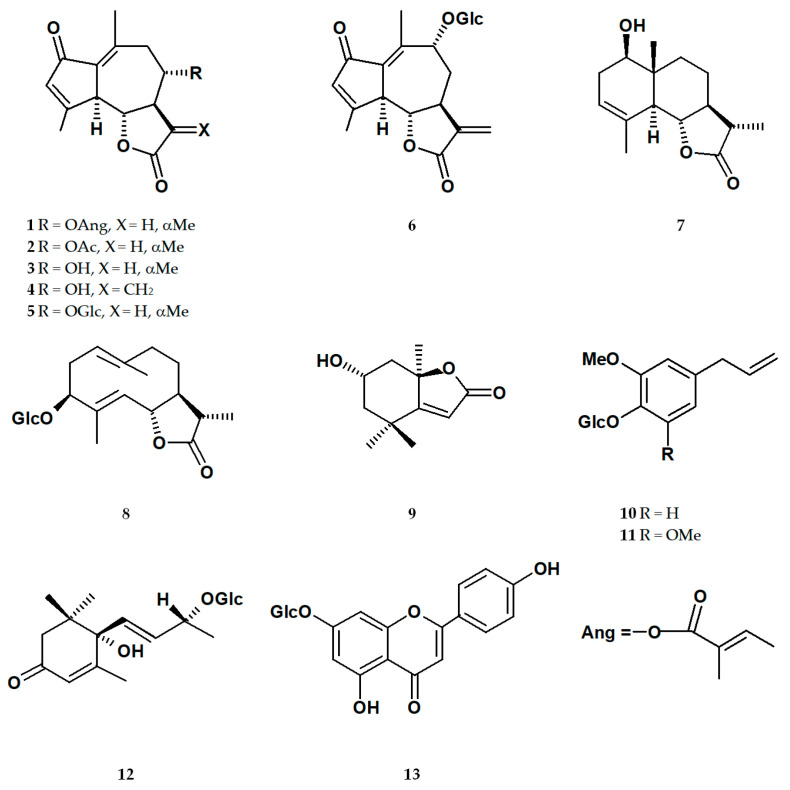
Chemical structures of the compounds isolated from the ethanolic extract of *Lactuca racemosa*: 8-*α*-angeloyloxyleucodin (**1**); matricarin (**2**); deacetylmatricarin (**3**); 15-deoxylactucin (11,13-dehydrodeacetylmatricarin) (**4**); deacetylmatricarin 8-*O*-*β*-glucopyranoside (**5**); 11,13-dehydrolactuside C (**6**); 11*β*,13-dihydrosantamarin (**7**); sonchuside A (**8**); loliolide (**9**); eugenyl 4-*O*-*β*-glucopyranoside (**10**); 5-methoxyeugenyl 4-*O*-*β*-glucopyranoside (**11**); (6*S*,9*S*) roseoside (**12**); apigenin 7-*O*-glucoside (**13**); Glc = *β*-glucopyranosyl.

From the ethanolic extracts of *L. racemosa* roots and aerial parts, eight sesquiterpenoids—8-*α*-angeloyloxyleucodin (**1**), matricarin (**2**), deacetylmatricarin (**3**), 15-deoxylactucin (11,13-dehydrodeacetylmatricarin; **4**), deacetylmatricarin 8-*β*-glucopyranoside (**5**), 11,13-dehydrolactuside C (**6**), 11*β*,13-dihydrosantamarin (**7**), and sonchuside A (**8**)—were isolated. Moreover, two apocarotenoids, loliolide (**9**) and (6*S*,9*S*) roseoside (**12**); two phenolics, eugenyl 4-*O*-*β*-glucopyranoside (**10**) and 5-methoxyeugenyl 4-*O*-*β*-glucopyranoside (**11**); and one flavonoid, apigenin 7-*O*-glucoside (**13**), were separated from the extracts (for structures, see Figure 1). Compounds **2**, **3**, and **5** were found in both the roots and aerial parts of the plant. Compounds **1**, **4**, **6**–**8**, and **10**–**11** derived from the roots, whilst **9** and **12–13** were obtained exclusively from the aerial parts.

### 2.2. RP-HPLC-PAD Analysis of Hydroalcoholic Extracts from L. racemosa and Semiquantitative Analysis of Selected Polyphenolic Constituents

**Figure 2:**
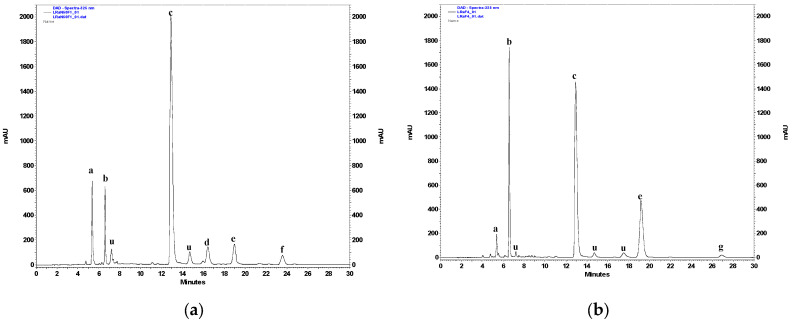
HPLC/PAD chromatogram of a hydroalcoholic extract from the aerial parts (**a**) and roots (**b**) of *Lactuca racemosa* (acquired at 325 nm): a—caftaric acid; b—chlorogenic acid; c—chicoric acid; d—luteolin 7-*O*-*β*-glucopyranoside; e—3,5-di-*O*-caffeoylquinic acid; f—apigenin 7-*O*-*β*-glucopyranoside; g—4,5-di-*O*-caffeoylquinic acid; u—unidentified caffeic acid derivative.

The chromatographic analysis, conducted according to the previously described procedure [17,18], disclosed compositions of the hydroalcoholic (70% methanol, *v*/*v*) extracts from the roots and the aerial parts of *L. racemosa* (Figure 2). Caffeic acid derivatives, including caftaric acid (CTA; t_R_ = 5.4 min), chlorogenic acid (5-CQA; t_R_ = 6.6 min), chicoric acid (DCTA; t_R_ = 12.9 min), and 3,5-di-O-caffeoylquinic acid (3,5-DCQA; t_R_ = 19.1 min), together with the flavonoids, luteolin 7-*O*-*β*-glucopyranoside (t_R_ = 16.4 min) and apigenin 7-*O*-*β*-glucopyranoside (t_R_ = 23.6 min), were identified as major constituents of the extracts from the aerial parts of the plant. Extracts from the roots did not contain flavonoids but did contain substantial amounts of DCTA, 3,5-DCQA, and 5-CQA. Minor amounts of CTA and 4,5-di-O-caffeoylquinic acid (4,5-DCQA, t_R_ = 26.9 min), as well as deacetylmatricarin 8-*O*-*β*-glucopyranoside (t_R_ = 11.1 min), were also detected. The contents of CTA, DCTA, 5-CQA, 3,5-DCQA, 4,5-DCQA, and luteolin 7-*O*-glucoside (see Table 1) were semiquantitatively estimated using the previously described method [17,18]. Chicoric acid dominated polyphenolic fraction in both the roots (1.18 ± 0.08% dry weight) and the aerial parts (3.00 ± 0.16%) of *L. racemosa*. The content of chlorogenic acid was similar in the roots (0.28 ± 0.08%) and the aboveground parts of the plant (0.23 ± 0.03%). Caftaric acid, like DCTA, was present in greater amounts (0.39 ± 0.04%) in the examined shoots rather than in the roots (0.06 ± 0.01%). Luteolin 7-*O*-glucoside constituted 0.45 ± 0.04% of the dry weight of the aerial parts of *L. racemosa*. Except for those visible on the chromatograms (Figure 2), the significant signals of phenolic compounds were not observed.

### 2.3. Measurements of the Reducing Capacity (TPC) and DPPH Radical Scavenging Activity of Extracts from L. racemosa

The reducing capacity of the plant material, often referred to as a “total phenolic content” (TPC), was measured using the Folin–Ciocalteu method. As expected, taking into consideration the contents of major phenolic constituents, the reducing capacity of the plant roots (12.96 ± 2.03 mg GAeq/g) was much lower than that of the aerial parts (58.42 ± 2.83 mg GAeq/g).

**Figure 3:**
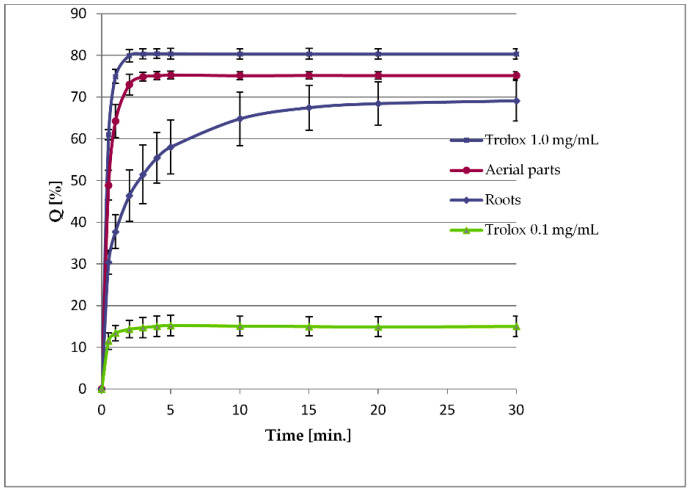
DPPH radical scavenging activity of Trolox (reference compound) and the hydroalcoholic extracts from the roots and aerial parts of *Lactuca racemosa* (10 mg of the dried plant material per 1 mL of extract).

DPPH radical scavenging measurements were performed to assess the radical-quenching activities of the extracts from *L. racemosa*. The experiments revealed a substantial difference in the activity of the examined extracts. After a 30 min reaction, the extract from roots scavenged 69.12 ± 4.86% of DPPH radical, and the extract from the aerial parts caused the quenching of 75.18 ± 0.87% of the available free radical. The extract from the aerial parts acted faster, and the reaction plateaued after just 5 min, whereas the extract from the roots demonstrated different kinetic reactions (Figure 3). 

### 2.4. Sesquiterpene Lactone Analysis

In the hydroalcoholic extract from the roots the signal of **5** (0.18 ± 0.07% of the dry plant material) was visible. For a more accurate assessment of the sesquiterpene lactone content, a series of samples was prepared following the standard protocol [19]. The analytical samples, prepared using MeOH as an extraction solvent, allowed for the estimation of matricarin (**2**), deacetylmatricarin (**3**), and deacetylmatricarin 8-*O*-*β*-glucopyranoside (**5**) content in the analyzed plant material (see Table 2, Supplementary Material Figure S14). 

### 2.5. Biosafety Assessment of Deacetylmatricarin in UN-SH-SY5Y and RA-SH-SY5Y Cells

**Figure 4:**
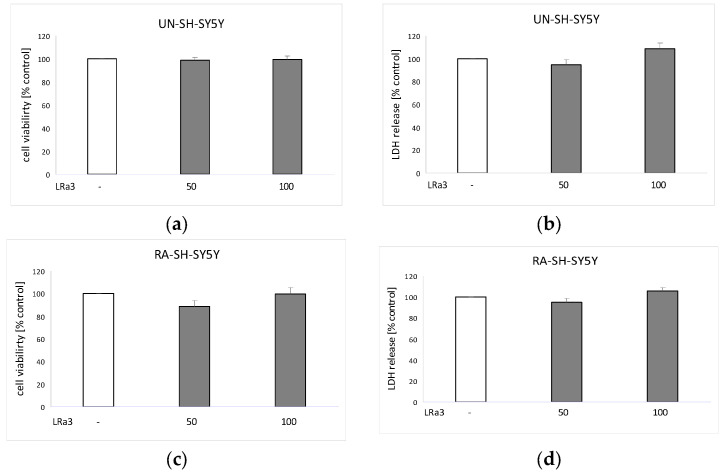
The impact of deacetylmatricarin (**3**, LRa3; 50 and 100 μM) on cell viability (**a**,**c**) and the cytotoxic activity of the compound (**b**,**d**) in UN-SH-SY5Y (**a**,**b**) and RA-SH-SY5Y (**c**,**d**) cells after a 24 h treatment. The data from MTT reduction assay (**a**,**c**) and LDH release assay (**b**,**d**) were normalized to the vehicle-treated cells and are presented as the mean ± SEM from four–six independent experiments with three–five replications.

As is shown in Figure 4, the tested compound, in the concentration range of up to 100 μM, did not affect the viability of the neuroblastoma cells in comparison to the vehicle-treated cells (control). 

### 2.6. The Effect of Deacetylmatricarin on H_2_O_2_- and 6-OHDA-Induced Cell Damage in UN-SH-SY5Y Cells

**Figure 5:**
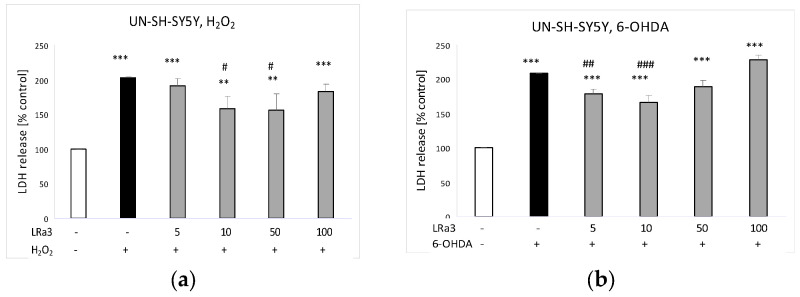
The protective effects of deacetylmatricarin (**3**, LRa3) against (**a**) hydrogen peroxide (H_2_O_2_)- and (**b**) 6-hydroxydopamine (6-OHDA)-evoked cell damage in UN-SH-SY5Y cells. The cell damaging effect was measured via LDH assay. Data were normalized to the vehicle-treated cells and are presented as the mean ± SEM from four independent experiments with three–five replications. ** *p* < 0.01 and *** *p* < 0.001 vs. the vehicle-treated cells; # *p* < 0.05, ## *p* < 0.01, and ### *p* < 0.001 vs. the H_2_O_2_- or 6-OHDA-treated cells.

The tested compound, in the concentration range of 10–50 μM, provided partial protection against the H_2_O_2_-caused injury in UN-SH-SY5Y cells (Figure 5a). In the model of the 6-OHDA-induced neurotoxicity, deacetylmatricarin demonstrated a moderate protective effect at concentrations of 5 and 10 μM (Figure 5b). 

### 2.7. The Effect of Deacetylmatricarin on H_2_O_2_- and 6-OHDA-Induced Cell Damage in RA-SH-SY5Y Cells

**Figure 6:**
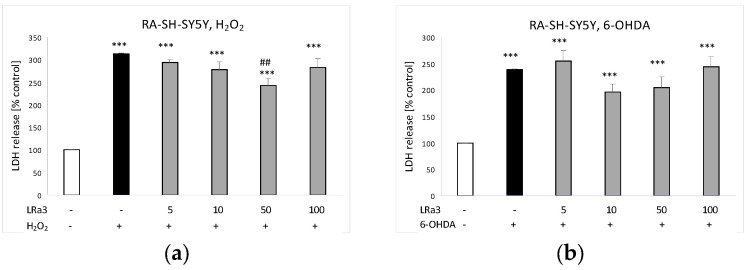
The effects of deacetylmatricarin (**3**, LRa3) against (**a**) hydrogen peroxide (H_2_O_2_)- and (**b**) 6-hydroxydopamine (6-OHDA)-evoked cell damage in RA-SH-SY5Y cells. The cell damaging effect was measured via LDH assay. Data were normalized to the vehicle-treated cells and are presented as the mean ± SEM from six independent experiments with three–five replications. *** *p* < 0.001 vs. the vehicle-treated cells; ## *p* < 0.01 vs. the H_2_O_2_-treated cells.

The investigated sesquiterpene lactone, at a concentration of 50 μM, moderately protected RA-SH-SY5Y cells against the H_2_O_2_-caused damage (Figure 6a). However, in the model of the 6-OHDA-induced toxicity, the protective effect of deacetylmatricarin was not observed (Figure 6b).

## 3. Discussion

During the course of the present study, guaianolides **1**–**2** and **4**–**5**, previously not known as metabolites of the *Lactuca* spp., were found in the roots and the aerial parts (**2** and **5**) of *L. racemosa*. Three compounds (**2**, **4**, and **5**) were obtained earlier from some other members of the tribe Cichorieae (Asteraceae family). To the best of our knowledge, compound **2** is only known from *Taraxacum bessarabicum* (Hornem.) Hand.-Mazz. [20], compound **4** from *Reichardia* spp. [12], while compound **5** (deacetylmatricarin 8-*O*-glucoside, notoserolide A) occurs in *Notoseris* spp. and *Taraxacum* spp. [12,13]. The occurrence of 8-*α*-angeloyloxyleucodin (austricin angelate, **1**) in the plants of the tribe Cichorieae has not been mentioned until now. The plant material also yielded a rare natural product, 11,13-dehydrolactuside C (**6**), which is known as a constituent of *Lactuca canadensis* L. [21], and deacetylmatricarin (austricin, **3**). This latter compound, though quite frequently described as a metabolite of plants from the Cichorieae tribe, has been isolated from the plant of the genus *Lactuca* only once before. An eudesmanolide, 11*β*,13-dihydrosantamarin (**7**), and a germacranolide, sonchuside A (**8**), were found in numerous taxa of the tribe [12,13].

Lactucin-type guaianolides (lactucin, 8-deoxylactucin, lactucopicrin) are the most characteristic and the best known terpenoid metabolites of the garden lettuce (*L. sativa*) and the *Lactuca* species included in its primary gene pool [7,12,13]. However, their absence from some other species of the genus is not unusual [13]. Sesquiterpene lactones isolated from *L. racemosa*, except for **7** and **8**, are lactucin-type guaianolides with a structure rarely seen in plants of *Lactuca* spp. The aerial parts, but especially the roots, of *L. racemosa* turned out to be a rich source of notoserolide A (**5**) (0.3% and 1.3%, respectively; see Table 2), a glucoside of deacetylmatricarin (**3**). The aglycone of **5** was accumulated mainly in the aboveground part of the plant (c. 0.05% dry weight). As the glucosides of the plant metabolites are usually rapidly hydrolyzed after the oral intake [22] and sesquiterpene lactone aglycones demonstrate higher biological activity than the corresponding glucosides [23], deacetylmatricarin (**3**) was further investigated with respect to its potential neuroprotective activity. The compound is a constituent of medicinally utilized plants (*Artemisia* spp., *Achillea* spp., and *Taraxacum* spp.) and its quantity and purity were sufficient to conduct the activity tests. 

Major phenolic metabolites of *L. racemosa* (CTA, 5-CQA, DCTA, 3,5-DCQA, luteolin 7-*O*-glucoside) are like those in the garden lettuce [17,24,25]. The most abundant compound, DCTA, constituted 3.00 ± 0.16% of the dried aboveground parts of *L. racemosa*. The contents of DCTA found in the garden lettuce ranged from 0.57 ± 0.06% dry weight in the “Kiribati” to 2.49 ± 0.25% dry weight in the “Gaugin” cultivars [17,24]. The remaining compounds were present in similar amounts in both *L. racemosa* and *L. sativa*. Apigenin 7-*O*-glucoside has not been described as a major constituent from *L. sativa* [24,25,26] but was isolated from several species of the genus *Lactuca* [14]. Moreover, in some metabolomic studies on the garden lettuce, the compound was tentatively identified using hyphenated analytical techniques [27,28]. The reducing capacity of the aerial parts of *L. racemosa* (58.42 ± 2.83 mg GAeq/g dry weight) was higher than that measured for the aerial parts of *L. sativa* (from 36.36 ± 2.78 to 46.81 ± 4.21 mg GAeq/g dry weight) [17] suggesting the presence of substantial amounts of antioxidative constituents in the plant material. The extract from the roots of *L. racemosa* was less effective in the DPPH scavenging experiment (Figure 3) than the extract from the aerial parts of the plant. This reflects the difference in polyphenols content (lower in roots of the plant, see Table 1). Antioxidative, anti-inflammatory, and neuroprotective activities of the hydroxycinnamic acid derivatives and flavonoids present in the lettuce leaves, as well as in the other vegetables and fruit, were the subject of numerous studies [29,30,31,32,33,34,35].

Deacetylmatricarin (austricin, **3**) is a constituent of *Taraxacum mongolicum* Hand.-Mazz. that is–at least in part–responsible for the anti-hepatocellular carcinoma activity of this plant extract as shown by the inhibition of HepG2 cell proliferation. The compound interacted with HSP90 proteins (heat shock proteins that aid protein folding and quality) and consequently regulated activation of protein kinase B (Akt) and phosphatidylinositol 3-kinase/protein kinase B (PI3K/Akt) signaling pathway associated with the tumor recurrence and drug resistance [36]. However, in our study we did not observe any effect of deacetylmatricarin (up to 100 μM) on the cell viability and cytotoxicity in both studied phenotypes of SH-SY5Y cells, which are of tumor origin (Figure 4). This very likely excludes this compound as a possible anti-tumor agent for neuroblastoma. In the antigen-evoked β-hexosaminidase release test, **3** inhibited degranulation of the basophilic leukemia cells (RBL-2H3 cell line) more potently than disodium cromoglycate [37]. The anti-inflammatory activity of **3** was investigated in vivo [38] and in vitro [39]. The compound inhibited the formalin-induced paw edema formation in mice and nitric oxide (NO) production in lipopolysaccharide (LPS)-stimulated murine macrophage (RAW 264.7) cells. 

In the current study, the neuroprotective activity of **3** against the H_2_O_2_- or 6-OHDA-induced oxidative stress-caused damage has been investigated using both undifferentiated (UN-SH-SY5Y) and retinoic acid-differentiated (RA-SH-SY5Y) neuroblastoma cells. The lactate dehydrogenase (LDH) and MTT tests proved that **3** did not show any cytotoxic activity towards the neuroblastoma cells at concentrations up to 100 μM (Figure 4b,d) which points to beneficial biosafety profile of this compound. Taking the results of our previous experiments into consideration, **3** was safer for the neuroblastoma cells than kaempferol 7-*O*-α-rhamnopyranoside (α-rhamnoisorobin) and methyl caffeate, which, at the concentrations of 50 μM and 50–100 μM, respectively, evoked cell damage in the UN-SH-SY5Y cells [40,41]. At the concentrations of 10 and 50 μM, **3** provided partial but statistically significant protection of the UN-SY-SY5Y cells against the H_2_O_2_-caused cell damage (Figure 5a). The 6-OHDA-evoked oxidative damage of the UN-SH-SY5Y cells was partially prevented by the pretreatment with 5 and 10 μM of **3** (Figure 5b). The protective effect of the compound was less pronounced in the retinoic acid-differentiated cells. At the concentration of 50 μM, deacetylmatricarin significantly diminished LDH release from the H_2_O_2_-treated RA-SH-SY5Y cells (Figure 6a) but was not effective against the 6-OHDA-induced cell death in this cell phenotype. 

Isoquercitrin, a flavonol of well known antioxidative and neuroprotective activity [42,43,44], was effective against the H_2_O_2_-evoked cell damage in both the UN-SH-SY5Y and RA-SH-SY5Y cells at the concentration of 50 μM. The protective effect of isoquercitrin against the 6-OHDA-induced oxidative injury in the UN-SH-SY5Y cells could be observed at the concentration of 10 and 50 μM, whereas in the differentiated neuroblastoma cells (RA-SH-SY5Y), isoquercitrin was less effective (only at the dose of 50 μM) [40].

Despite the differences in the chemical structure, in the experimental model applied in the present study, **3** demonstrated neuroprotective activity like polyphenolic compounds. It is not excluded that the regulation of PI3K/Akt signaling pathway [36] may be implicated in the cell protection mediated by **3**. It could be supported by the fact that during the retinoic acid-induced SH-SY5Y cell differentiation, an activation of various intracellular pro-survival pathways, e.g., PI3-K/Akt [45] has been evidenced. The activation could attenuate or mask the potential protective effects of **3** as observed by us in the RA-SH-SY5Y cells exposed to H_2_O_2_ or 6-OHDA. It should be noted that in our study, we observed higher resistance of the RA-SH-SY5Y cells to the cytotoxic action of H_2_O_2_ or 6-OHDA as evidenced by higher concentrations of the cell damaging factors used (0.375 and 0.5 mM H_2_O_2_ in UN-and RA-SH-SY5Y cells, respectively; 100 and 200 μM of 6-OHDA in UN-and RA-SH-SY5Y cells, respectively) to induce a similar degree of cell damage (about 50% reduction in cell viability). This was also confirmed in our previous reports [40,41]. Moreover, in our previous study we showed that the neuroprotection mediated by α-rhamnoisorobin or isoquercitrin against the H_2_O_2_-induced cell damage in the UN-SH-SY5Y cells was inhibited by PI3-K pathway inhibitor, LY294002 [40]. However, the detailed mechanism of the cytoprotective action of deacetylmatricarin needs further studies.

## 4. Materials and Methods

### 4.1. Chemicals and Solvents

Standard samples of caffeoylquinic acids (5-CQA, DCTA, CTA, 1,3-DCQA; declared purity 97–99%), luteolin 7-O-β-D-glucoside (purity ≥ 98%). and gallic acid (GA) were purchased from Roth (Karlsruhe, Germany) and Sigma-Aldrich Co. (St. Louis, MO, USA). Trolox (6-hydroxy-2,5,7,8-tetramethylchroman-2-carboxylic acid), Folin–Ciocalteu reagent, and DPPH (2,2-diphenyl-1-picrylhydrazyl) were supplied by Sigma-Aldrich Co. Apigenin 7-*O*-*β*-glucoside and 3,5-DCQA were obtained from different plants of the Cichorieae tribe and identified by comparison of their spectral data with those found in the literature. 

Organic solvents CHCl_3_, ethyl acetate (EtOAc), and MeOH (analytical grade) were bought from Avantor Performance Materials Poland S.A. (Gliwice, Poland). The Mili-Q system (Millipore Corp., Bedford, MA, USA) was used to obtain purified water. HPLC-grade MeOH and acetonitrile (MeCN), together with analytical-grade acetic acid (CH_3_COOH), formic acid (HCOOH), and n-hexane, were supplied by Merck (Darmstadt, Germany).

Media and supplements for the neuroblastoma cell culture, i.e., Dulbecco’s modified eagle medium (DMEM), trypsin/EDTA (0.25%) solution, heat-inactivated fetal bovine serum (FBS), and a mixture of penicillin and streptomycin, were bought from Gibco (Invitrogen, Paisley, UK). The Cytotoxicity Detection Kit (LDH release assay) manufactured by Roche Diagnostic (Mannheim, Germany) was used in the experiments. Stabilized hydrogen peroxide solution (30% H_2_O_2_), 6-OHDA hydrochloride, retinoic acid (RA), and MTT were supplied by Sigma-Aldrich (Sigma-Aldrich Chemie GmbH, Taufkirchen, Germany).

### 4.2. General Experimental Procedures

A Bruker AVANCE III 400 (400.17 MHz for ^1^H and 100.63 MHz for ^13^C) spectrometer (Bruker Corp., Billerica, MA, USA) was used to perform NMR experiments. The spectra were recorded in CDCl_3_, MeOD, or in pyridine-d_5_. A Waters instrument (Waters Corp., Milford, MA, USA), coupled to a dual wavelength UV/VIS detector and Delta-Pak C-18 column (Waters Corp., Milford, MA, USA; particle size 15 μm, 25 × 100 mm), was employed to conduct semipreparative RP-HPLC. H_2_O-MeOH mixtures, at a flow rate of 3.0 mL/min, were used as eluents.

Analytical RP-HPLC separations of phenolic compounds were conducted at 25 °C on a Zorbax Eclipse XDB-C18 column, 4.6 × 150 mm (Agilent Technologies, Santa Clara, CA, USA). Sesquiterpene lactones were separated at 40 °C on a Kinetex XB-C18 column (4.6 × 250 mm, 5 μm; Phenomenex, Torrance, CA, USA). An Agilent 1200 Series HPLC system (Agilent Technologies, Santa Clara, CA, USA) equipped with a photodiode array detector (PAD) was used to perform analyses. 

Merck silica gel 60 (0.063–0.2 mm) and precoated Merck silica gel 60 (0.25 mm) plates (Merck, Darmstadt, Germany) were used for the conventional column chromatography (CC) and thin-layer chromatography separations, respectively. After the TLC separations, the analyzed compounds were detected by viewing under UV light, as well as by spraying with 20% sulfuric acid and heating.

### 4.3. Plant Material

Botanically verified L. racemosa seeds (IPEN GE-0-BONN-18479), originating from Georgia (Khevi region), were supplied by the Botanical Gardens of the University of Bonn (Germany). The seeds were germinated, and the plants of L. racemosa were grown in the Garden of Medicinal Plants of the Maj Institute of Pharmacology, Polish Academy of Sciences, Kraków, Poland (voucher specimen 8/2019). The taxonomic identity of the plants was confirmed by one of the authors (WP). The roots and aerial parts of L. racemosa were collected in the flowering phase, on 25 June 2020, in the second year of cultivation.

### 4.4. Extraction and Isolation

The dried and pulverized plant materials (39 g of roots and 66 g of aerial parts) were separately extracted with 96% EtOH (*v*/*v*) (7 × 400 mL and 7 × 600 mL, respectively) at a room temperature by shaking. EtOH was evaporated in vacuo to give 8.0 g of the crude extract from the roots and 16.0 g of the crude extract from the aboveground parts. The extracts were subsequently chromatographed, separately, on silica gel columns eluted with n-hexane-EtOAc gradient solvent system (up to 100% EtOAc), followed by EtOAc-MeOH (up to 10% MeOH). The obtained fractions were surveyed by analytical RP-HPLC and TLC. Further separations were conducted using semipreparative RP-HPLC.

The fractions from roots, eluted with hexane-EtOAc (4:1, *v*/*v*), were subjected to RP-HPLC (H_2_O-MeOH, 1:4, *v*/*v*) to yield **1** (6.9 mg). The fractions eluted with hexane-EtOAc (7:3, *v*/*v*) gave **2** (2.6 mg) and **7** (3.9 mg), after separation by RP-HPLC (H_2_O-MeOH, 2:3, *v*/*v*). Compounds **3** (6.0 mg) and **4** (1.0 mg) were obtained by the elution of the silica gel column with hexane-EtOAc (3:2, *v*/*v*) and subsequent separation of the eluted fractions by RP-HPLC (H_2_O-MeOH, 1:1, *v*/*v*). Fractions from the EtOAc elution were further purified by RP-HPLC (H_2_O-MeOH 1:1, *v*/*v*) to give **5** (26.3 mg) and a mixture of **8**, **10**, and **11** (ca. 1:1.5:2, 2.1 mg, according to ^1^H NMR). The fractions eluted with EtOAc-MeOH (49:1, *v*/*v*), after the RP-HPLC separation (H_2_O-MeOH 3:2, *v*/*v*) yielded **6** (4.3 mg).

The crude extract from *L. racemosa* aerial parts was fractionated, as described above, to give **2** (6.9 mg), **3** (12.7 mg), and **5** (18.9 mg). The fractions eluted from the silica gel column with n-hexane-EtOAc (7:3) and further separated by RP-HPLC (H_2_O-MeOH, 3:7) afforded **9** (4.6 mg). The fractions eluted with EtOAc, after the RP-HPLC separation (H_2_O-MeOH, 3:2), gave **12** (2.4 mg) and **13** (4.2 mg).

Compounds **2**–**3** and **5**–**13** were identified by comparing chromatographic behavior and spectroscopic data (UV-VIS, ^1^H NMR) of the isolates with those of the compounds obtained previously in our lab from *Taraxacum* spp. and *Lactuca* spp. [14,17,18,20,21]. Compounds **1** and **4**, derivatives of deacetylmatricarin, were identified by comparison of their spectral data (^1^H NMR) with those found in the literature [46,47] and with those of the previously isolated deacetylmatricarin derivatives.

### 4.5. Identification of Major Phenolic Constituents from Roots and Aerial Parts of L. racemosa and Estimation of Their Contents

The dry and pulverized roots (100 mg) or aerial parts (50 mg) of *L. racemosa* were extracted with 10 mL of 70% (*v*/*v*) MeOH (2 × 2 h) at a room temperature using a rotary shaker. Then, the extracts from the two subsequent extractions were combined and further processed, as described elsewhere [17]. Chromatographic separations of phenolic compounds were performed as before [17,18]. Peak areas were measured either at 325 nm (hydroxycinnamates) or at 345 nm (luteolin 7-*O*-*β*-glucoside). Quantification was conducted via an external standard method [17,18].

### 4.6. Measurement of the Reducing Capacity of the Plant Material (TPC)

The plant material under study (10 mg) was extracted with 2 mL of 80% MeOH containing 1% HCl (2 × 2 h) using a reciprocal shaker. The combined extracts were analyzed as described previously [17]. The results were expressed as gallic acid equivalents (mg) per gram of the dry plant material (mg GAeq/g DW).

### 4.7. DPPH Radical Scavenging Assay

The dried and pulverized plant material (100 mg) was extracted with 12.5 mL of 50% MeOH (2 × 2 h) at a room temperature. The extracts were combined and evaporated to dryness. The obtained residue was dissolved in 70% MeOH (1 mL), left to stand overnight at 4 °C, and subsequently centrifuged (11,340× *g*, 5 min). The supernatant was diluted 10 times to obtain the concentration corresponding to 10 mg DW of plant material/1 mL. The radical scavenging assay was performed as described earlier [17]. A decrease in the absorbance (λ = 517 nm) was measured with a UV/VIS CE 2021 spectrophotometer (Cecil, Cambridge, UK).

### 4.8. Measurement of the Matricarin Derivatives Content

Analytical samples, prepared via the extraction of the dry and pulverized plant tissues (200 mg) with MeOH, were subjected to RP-HPLC/DAD analysis, as described previously [19]. In the samples, sesquiterpene lactones were identified by their RP-HPLC retention times and HPLC–PAD spectra, with a distinctive chromophore (conjugated π bond system; λmax = 260 nm), in comparison with the standard samples of matricarin (**2**, t_R_ = 29.3 min), deacetylmatricarin (**3**, t_R_ = 23.9 min), and deacetylmatricarin 8-*O*-*β*-glucopyranoside (**5**, t_R_ = 12.8 min), isolated earlier from *L. racemosa*. The quantification was conducted via the external standard method using the standard curve derived from five concentrations (0.005 to 1.000 mg/mL) of **5**. Peak areas were measured at 260 nm. Every analysis was conducted in triplicate (samples were derived from three different plants).

### 4.9. SH-SY5Y Cell Culture

The human SH-SY5Y neuroblastoma cells (ATCC CRL-2266, Manassas, VA, USA; passages 4–15) were cultured in T75cm2 cell culture flasks in DMEM with the addition of 10% FBS and 1% penicillin–streptomycin solution. The cells were maintained at 37 °C under conditions of saturated humidity in an atmosphere containing 95% air and 5% CO_2_. The confluent cells (~80%) were trypsynized (0.05% trypsin/EDTA solution at room temperature), manually counted (Bürker chamber), and seeded into 96-well-plates at a density of 4 × 10^4^ and 2 × 10^4^ cells per well for the UN-SH-SY5Y and RA-SH-SY5Y cells, respectively. The RA-SH-SY5Y cells were cultured in a cell culture medium supplemented with 10 μM RA for 6 days, as described previously [41]. To limit cell proliferation, one day prior to the experiments, the culture medium for the UN-SH-SY5Y and RA-SH-SY5Y cells was replaced with DMEM containing 1% FBS and 1% penicillin–streptomycin solution.

### 4.10. Cell Treatment

First, the biosafety assessment of **3** was conducted in the UN-SH-SY5Y and RA-SH-SY5Y cells treated for 24 h with **3** (50 and 100 μM). The neuroprotective potency of **3** was investigated in the UN- and RA-SH-SY5Y cells, which were pretreated for 30 min with different concentrations of **3** (5, 10, 50, and 100 μM), followed by 24 h exposure to H_2_O_2_ (0.375 mM and 0.5 mM for UN- and RA-SH-SY5Y cells, respectively) or 6-OHDA (100 μM and 200 μM for UN- and RA-SH-SY5Y cells, respectively). The effective concentrations of the cell damaging factors (H_2_O_2_, 6-OHDA) were established in our previous studies where these factors reduced cell viability by about 50% [45]. The stock solution of **3** (5 mM) was prepared in 70% ethanol, aliquoted, and stored at −20 °C. The H_2_O_2_ (25 and 50 mM) stock solutions were prepared from stabilized 30% H_2_O_2_ diluted in distilled water. The 6-OHDA (10 and 20 mM) stock solutions were prepared in distilled water immediately before use. All agents were added to the experimental plates under limited light. The control cultures were supplemented with the appropriate vehicles, and the solvent was present in the cultures at a final concentration of 0.1%.

### 4.11. Cell Viability Assay

The cell viability was measured via MTT assay, as described previously [42]. The absorbance of the probes was measured at 570 nm with a microplate reader: Infinite® M200 PRO (Tecan Austria GmbH, Grödig, Austria).

### 4.12. Cytotoxicity Assay (LDH Release Assay)

The level of LDH released into culture media after the cell treatments was measured with the Cytotoxicity Detection Kit (Roche Diagnostic), as described previously [42]. The absorbance of the probes was measured at 490 nm with a microplate reader: Infinite® M200 PRO (Tecan Austria GmbH, Grödig, Austria).

### 4.13. Statistical Analysis

The biochemical data, after blanks subtractions and normalizations, were statistically analyzed with the Statistica 13 software (StatSoft Inc., Tulsa, OK, USA) with an assumed *p* < 0.05. The one-way ANOVA and post hoc Duncan’s test for multiple comparisons were used.

## 5. Conclusions

The present study revealed major terpenoid and phenolic metabolites synthesized by *L. racemosa*, a species of wild lettuce that has not been phytochemically investigated before. The plant is rich in matricarin derivatives, which is unusual in *Lactuca* spp. Also worthy of attention is the high content of chicoric acid (3% dry weight). Phenolic compounds accumulated by the plant’s aerial parts corresponded to those found in the garden lettuce. In addition to the commonly known polyphenolic antioxidants, *L. racemosa* accumulated deacetylmatricarin and its glucoside. The sesquiterpene lactone demonstrated protective activity towards the H_2_O_2_- and 6-OHDA-induced oxidative damage in human neuroblastoma cells. The chemical data from our study may be used to establish chemophenetic relationships in the genus *Lactuca*. Our data may also support the usage of aerial parts of *L. racemosa* as a functional food due to the presence of compounds with potential health-enhancing properties.

## Figures and Tables

**Table 1:** Contents of the major hydroxycinnamates and luteolin 7-*O*-glucoside in *Lactuca racemosa* roots and aerial parts.

*Lactuca racemosa*	Phenolic Constituents (% Dry Weight) ^1^					
	CTA	5-CQA	DCTA	3,5-DCQA	4,5-DCQA	Luteolin 7-*O*-*β*-glucoside
Aerial parts	0.39 ± 0.04	0.23 ± 0.03	3.00 ± 0.16	0.28 ± 0.09	nd	0.45 ± 0.04
Roots	0.06 ± 0.01	0.28 ± 0.08	1.18 ± 0.08	0.45 ± 0.08	0.020 ± 0.004	nd

^1^ The results are means of three independent measurements (three individual plants) ± SD; nd—not detected.

**Table 2:** Contents of the matricarin derivatives **2**, **3**, and **5** in the roots and aerial parts of *Lactuca racemosa*.

*Lactuca* *racemosa*	Sesquiterpene Lactones (% Dry Weight) ^1^		
	Matricarin (2)	Deacetylmatricarin (3)	Deacetylmatricarin 8-*O*-*β*-glucopyranoside (5)
Aerial parts	0.010 ± 0.008	0.046 ± 0.020	0.326 ± 0.162
Roots	0.033 ± 0.011	0.007 ± 0.002	1.260 ± 0.572

^1^ The results are the means of three independent measurements (three individual plants) ± SD.

## Data Availability

Data are contained within the article or Supplementary Materials.

## Supplementary Materials

The following supporting information can be downloaded at https://www.mdpi.com/article/10.3390/molecules29245975/s1; Figures S1–S14: ^1^H NMR spectra of compounds **1**–**8**; ^13^C MNR spectra of compounds **2**, **3**, **5**–**7**; HPLC/PAD chromatogram of a methanol extract from roots of *Lactuca racemosa*.
