# Supplementary material for: Lactuca racemosa Willd., Source of Antioxidants with Diverse Chemical Structures
Source: Molecules. 2024 Dec 18;29(24):5975. doi: 10.3390/molecules29245975 (PMC11676871; doi:10.3390/molecules29245975)
Supplement: Supplementary file 1 [file molecules-29-05975-s001.zip › molecules-3312672-supplementary.pdf]

Lactuca racemosa Willd., Source of Antioxidants with Diverse Chemical Structures.

Klaudia Michalska, Danuta Jantas, Janusz Malarz, Klaudia Jakubowska, Wojciech Paul, and Anna Stojakowska

SUPPLEMENTARY MATERIAL

Figure S1, Figure S1a, Figure S1b, Figure S1c. ^{1}H NMR spectrum of compound **1** in acetone-d_6.

Figure S2, Figure S2a, Figure S2b. ^{1}H NMR spectrum of compound **2** in $CDCl_3$.

Figure S3. ^{13}C NMR spectrum of compound **2** in $CDCl_3$.

Figure S4, Figure S4a, Figure S4b. ^{1}H NMR spectrum of compound **3** in $CDCl_3$.

Figure S5. ^{13}C NMR spectrum of compound **3** in $CDCl_3$.

Figure S6, Figure S6a, Figure S6b. ^{1}H NMR spectrum of compound **4** in $CDCl_3$.

Figure S7, Figure S7a, Figure S7b. ^{1}H NMR spectrum of compound **5** in pyridine-d_5.

Figure S8. ^{13}C NMR spectrum of compound **5** in pyridine-d_5.

Figure S9, Figure S9a, Figure S9b. ^{1}H NMR spectrum of compound **6** in pyridine-d_5.

Figure S10. ^{13}C NMR spectrum of compound **6** in pyridine-d_5.

Figure S11, Figure S11a, Figure S11b. ^{1}H NMR spectrum of compound **7** in $CDCl_3$.

Figure S12. ^{13}C NMR spectrum of compound **7** in $CDCl_3$.

Figure S13, Figure S13a, Figure S13b. ^{1}H NMR spectrum of compound **8** in pyridine-d_5.

Figure S14. HPLC/PAD chromatogram of a methanol extract from the roots of *Lactuca racemosa* (acquired at 260 nm): **5** – deacetylmatricarin 8-*β*-glucopyranoside; **6** – 11,13-dehydrolactuside C; **3** – deacetylmatricarin; **2** – matricarin.

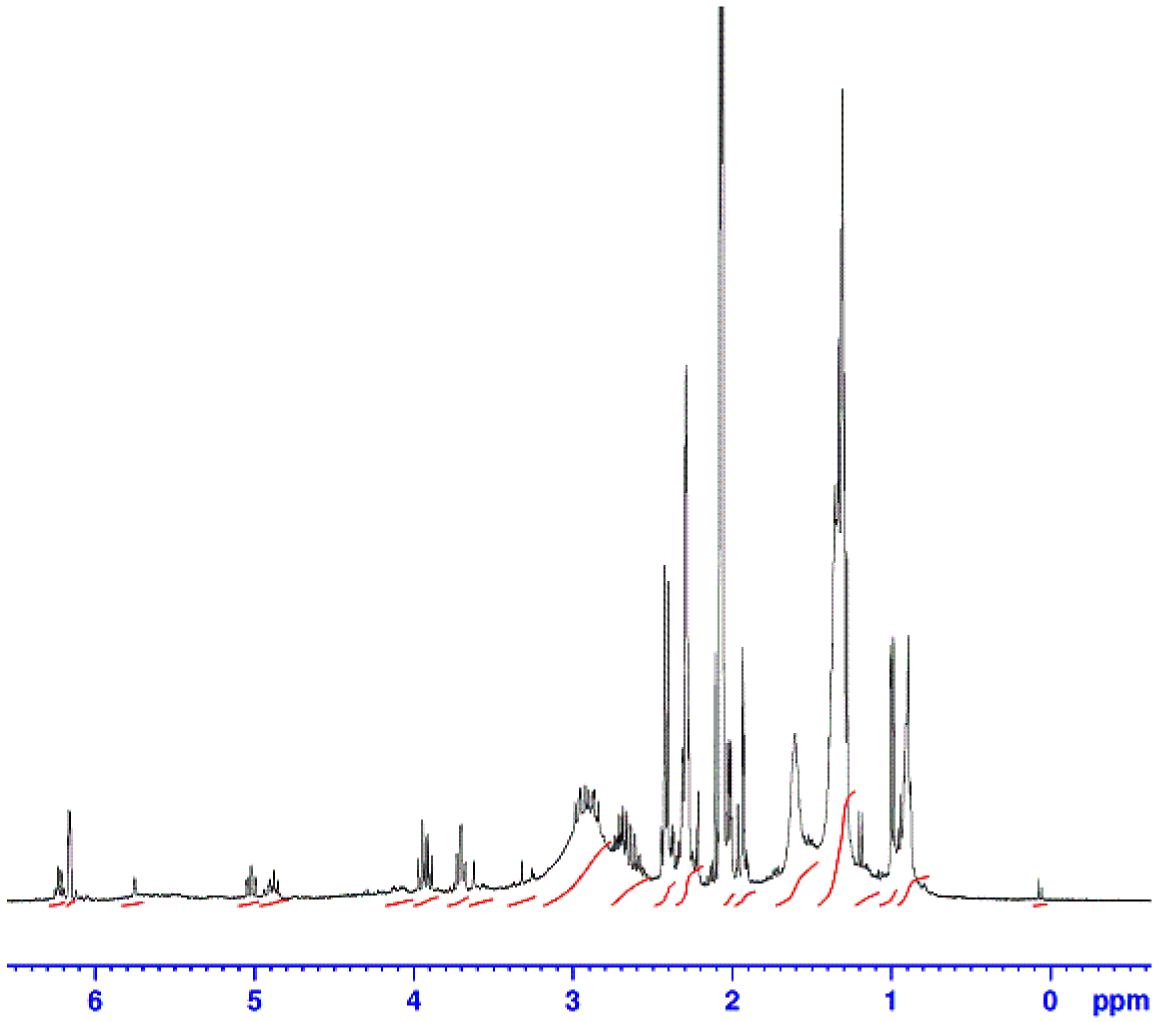

Figure S1. ^{1}H NMR spectrum of compound **1**.

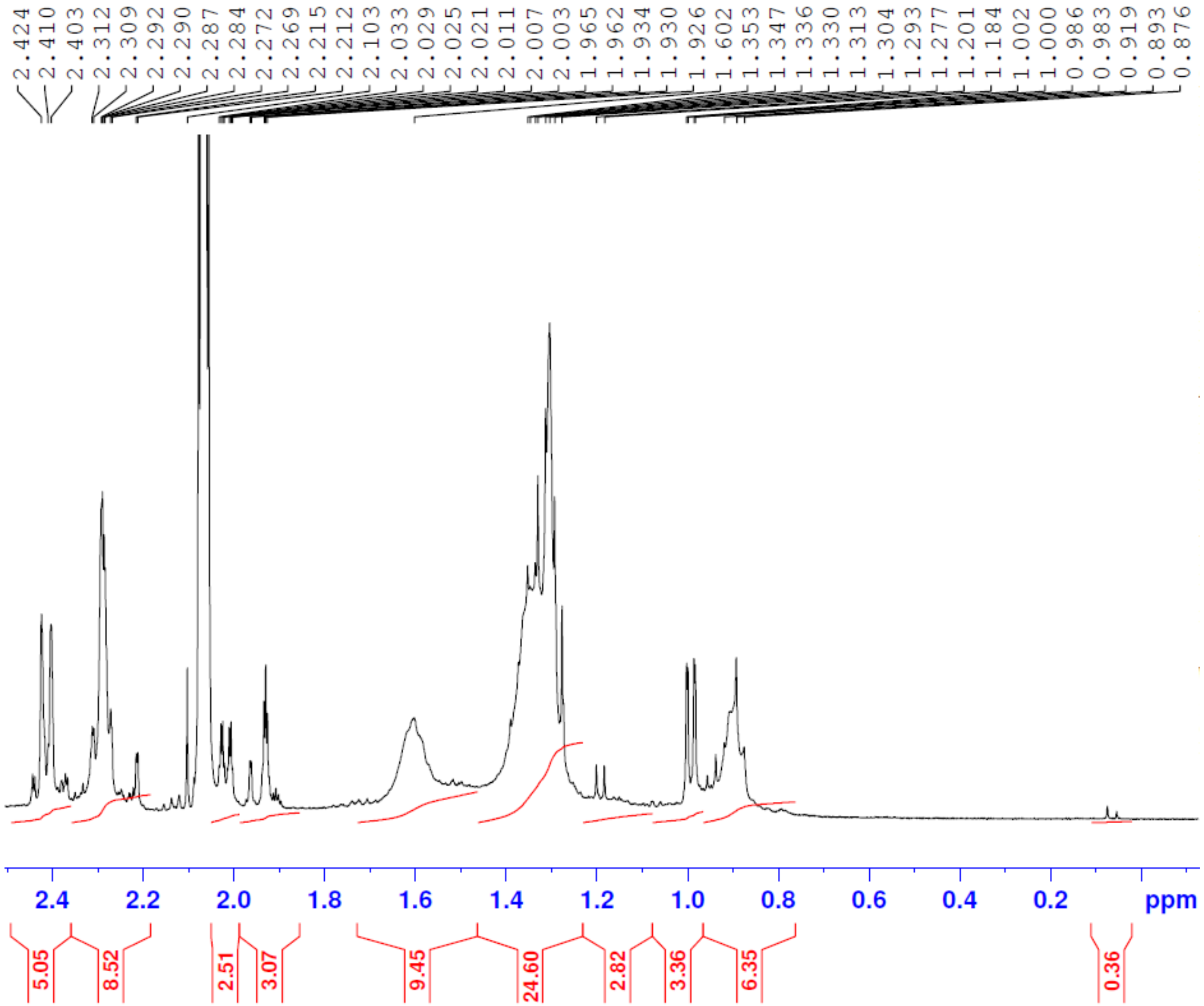

Figure S1a. ^{1}H NMR spectrum of compound **1**.

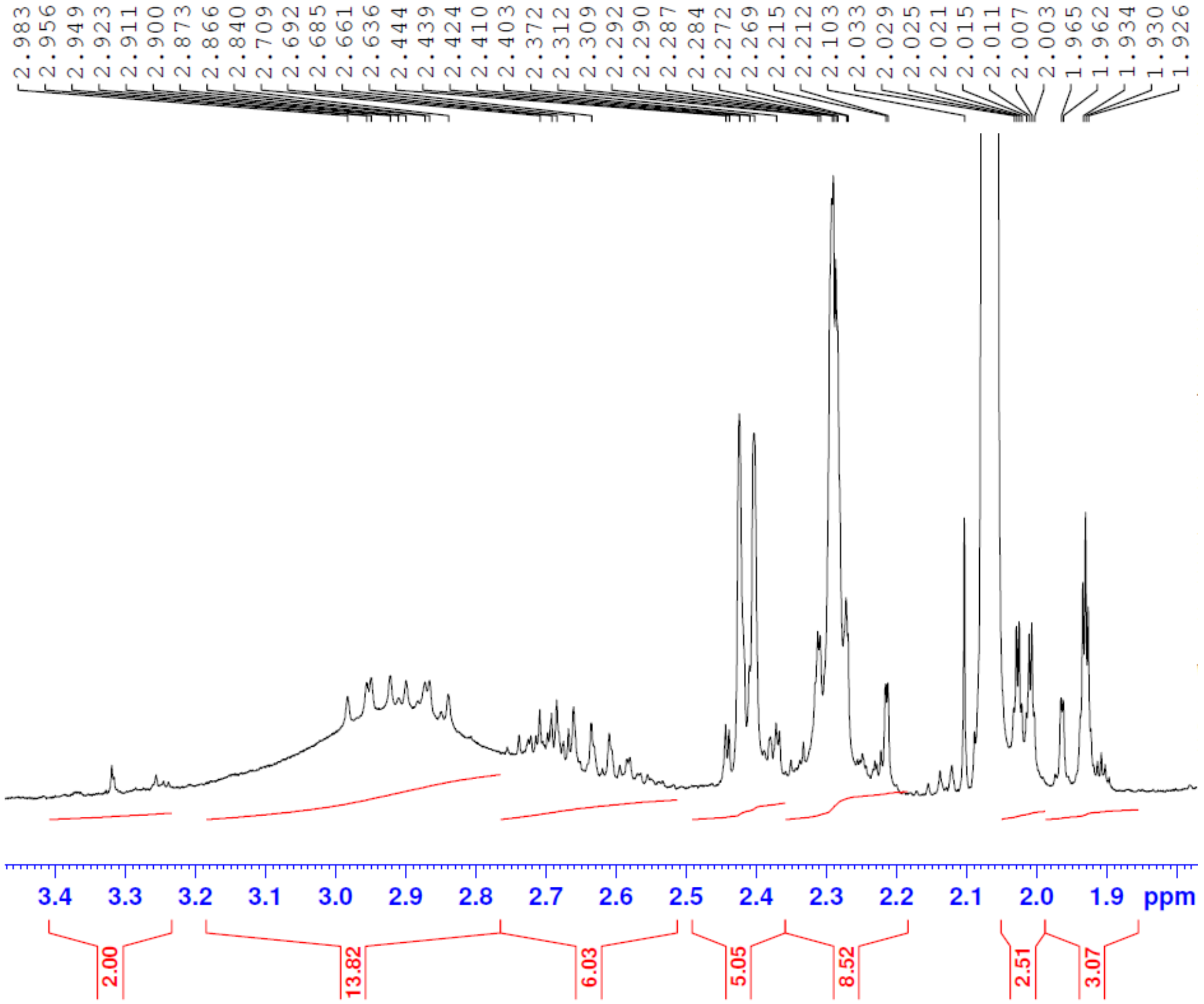

Figure S1b. ^{1}H NMR spectrum of compound **1**.

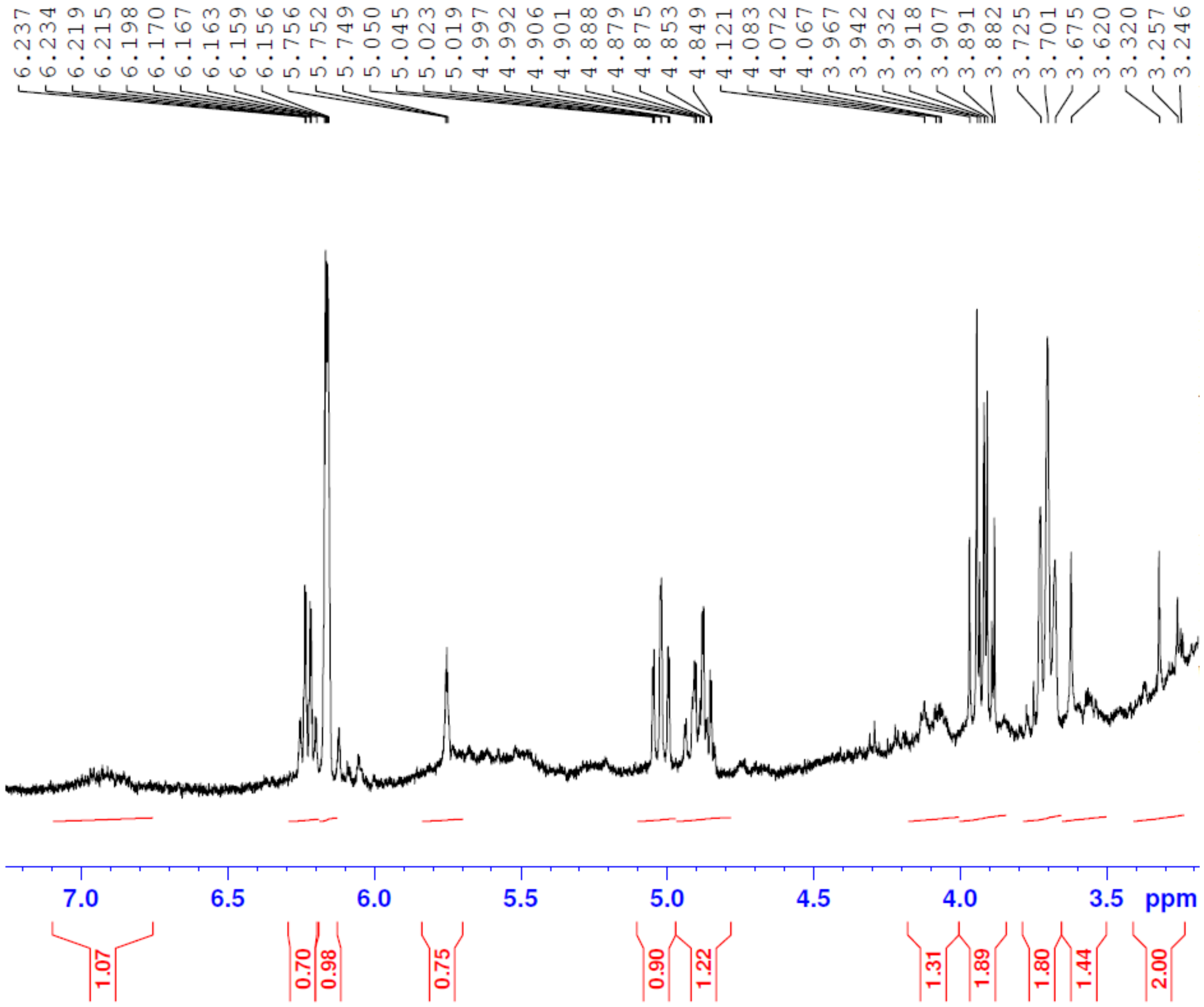

Figure S1c. ^{1}H NMR spectrum of compound **1**.

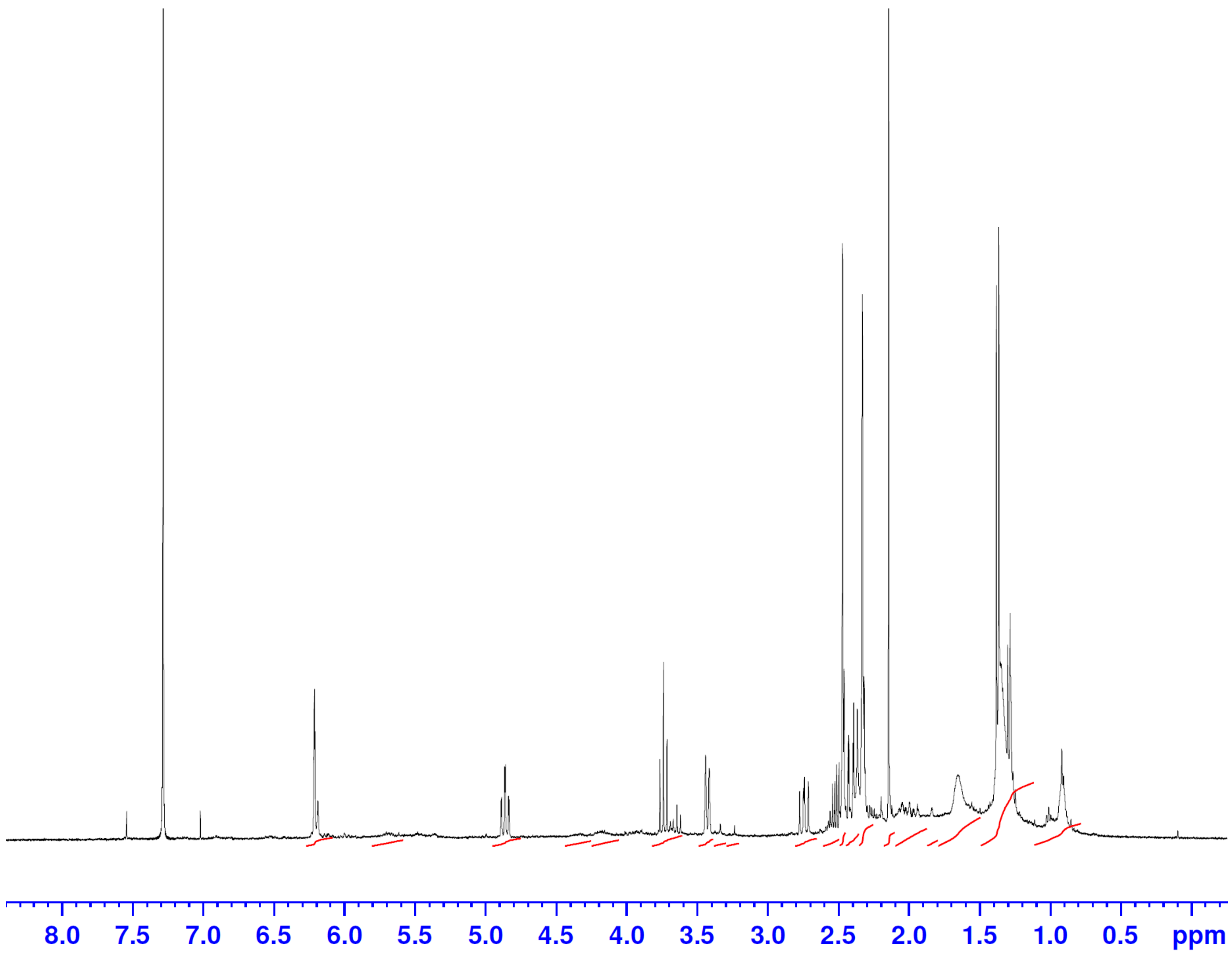

Figure S2. ^{1}H NMR spectrum of compound **2**.

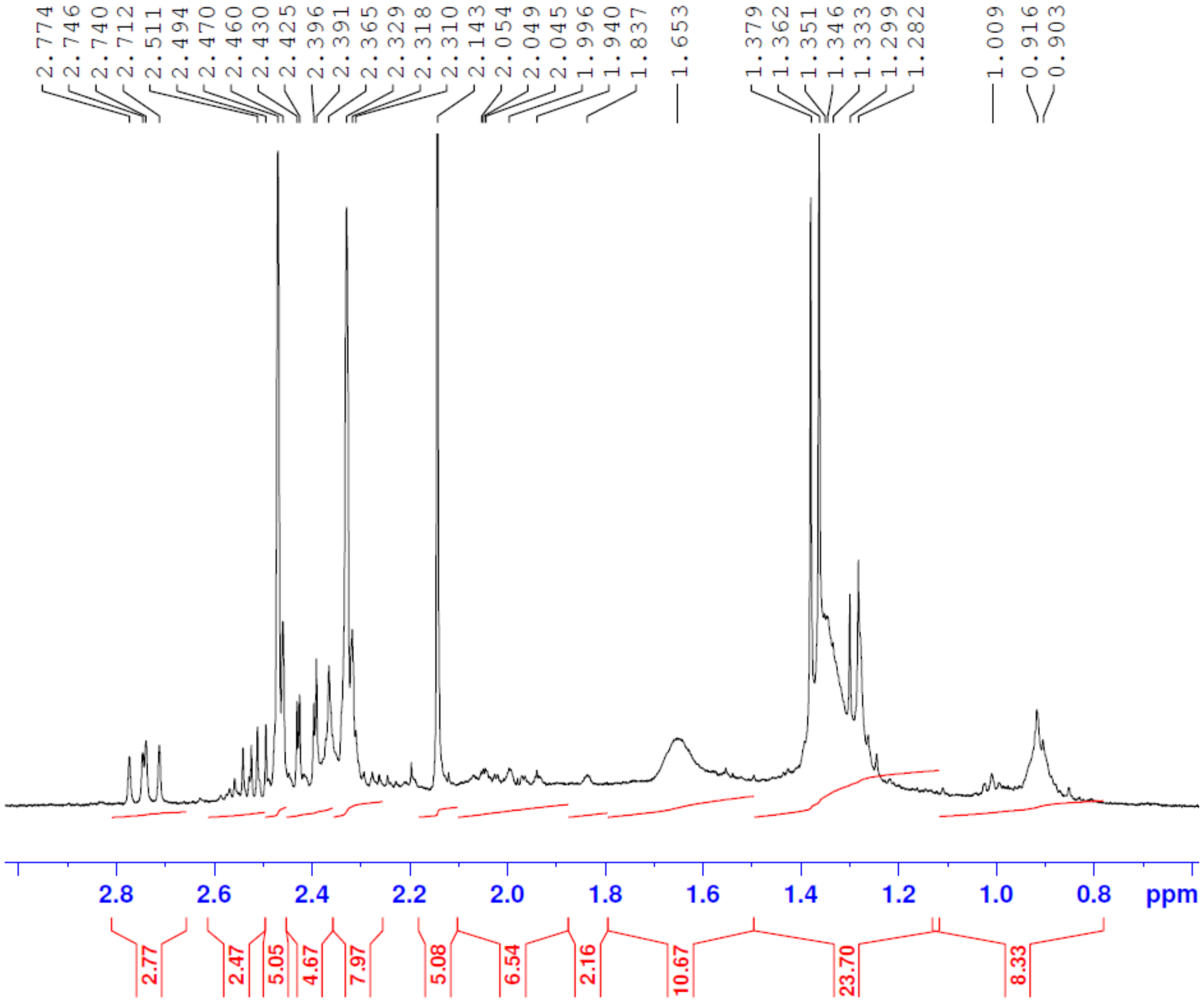

Figure S2a. ^{1}H NMR spectrum of compound **2**.

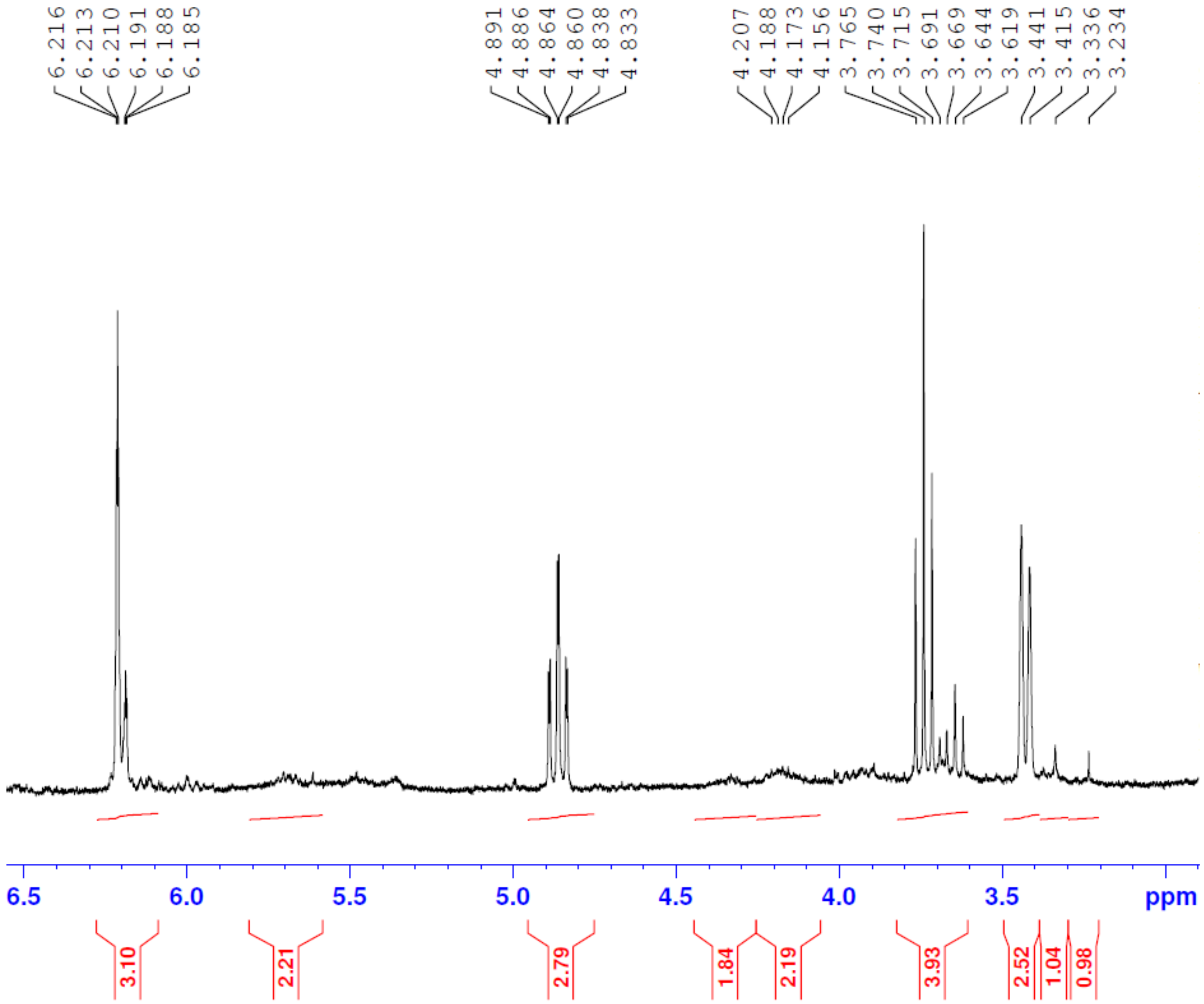

Figure S2b. ^{1}H NMR spectrum of compound **2**.

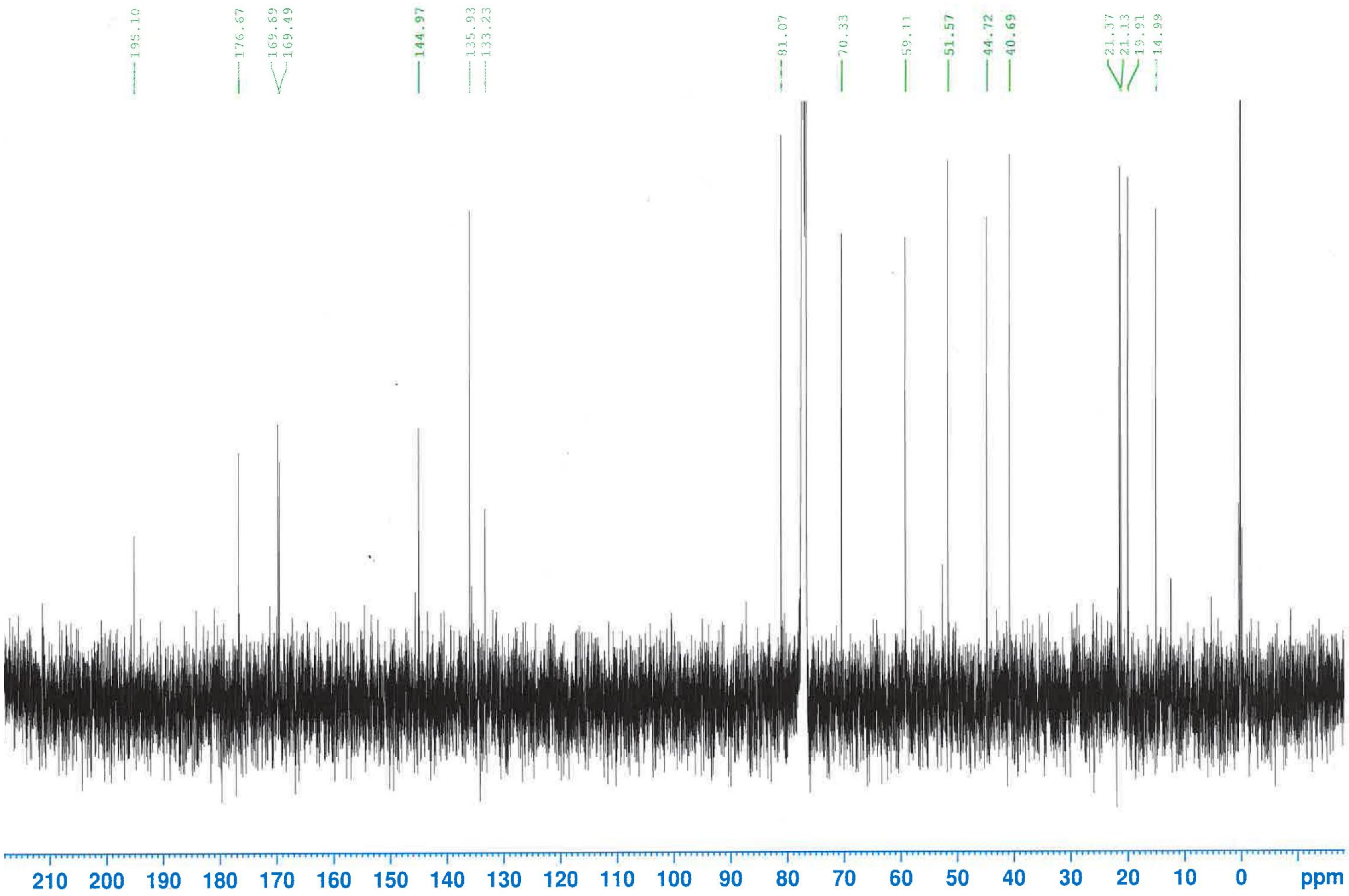

Figure S3. ^{13}C NMR spectrum of compound **2**.

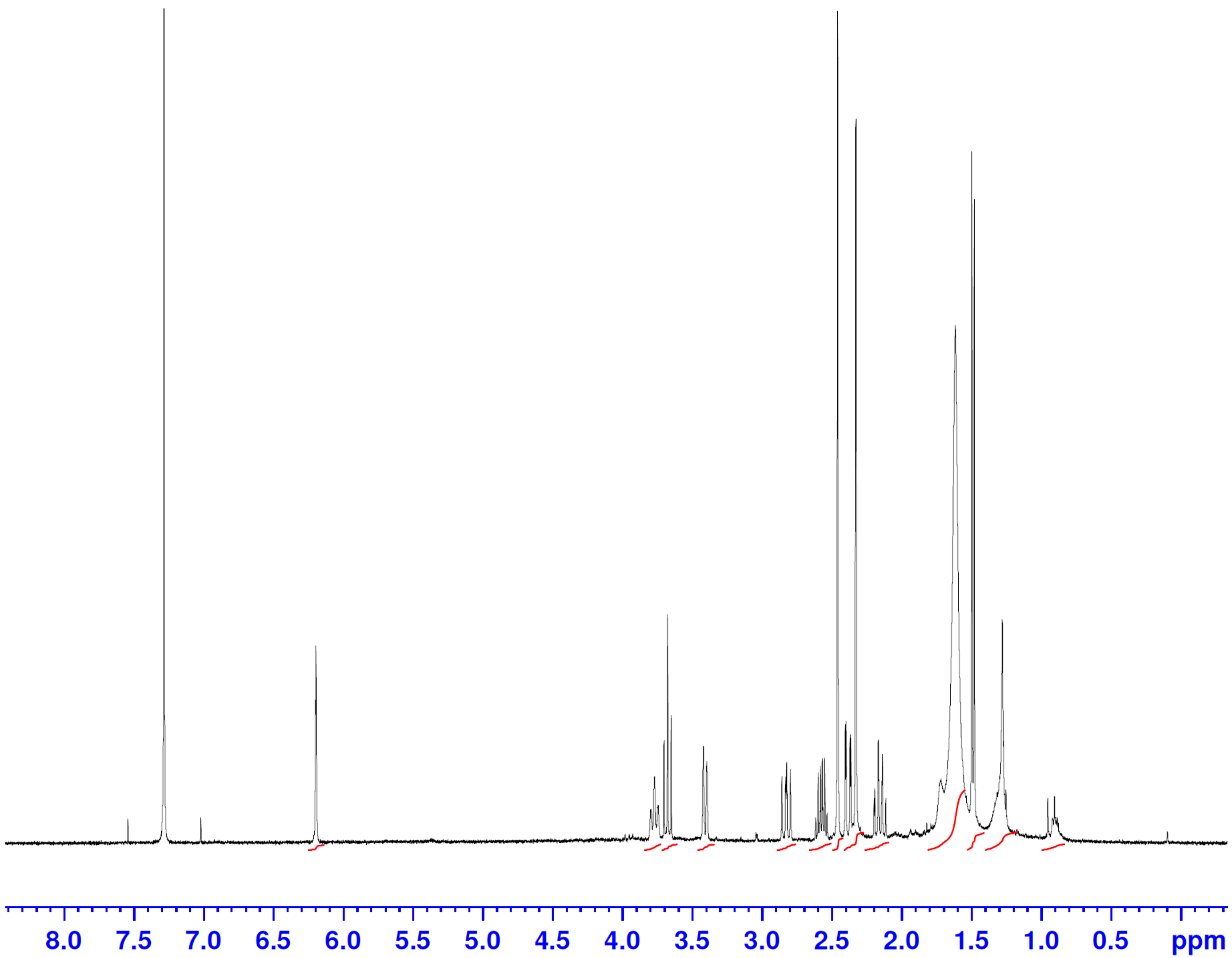

Figure S4. ^{1}H NMR spectrum of compound **3**.

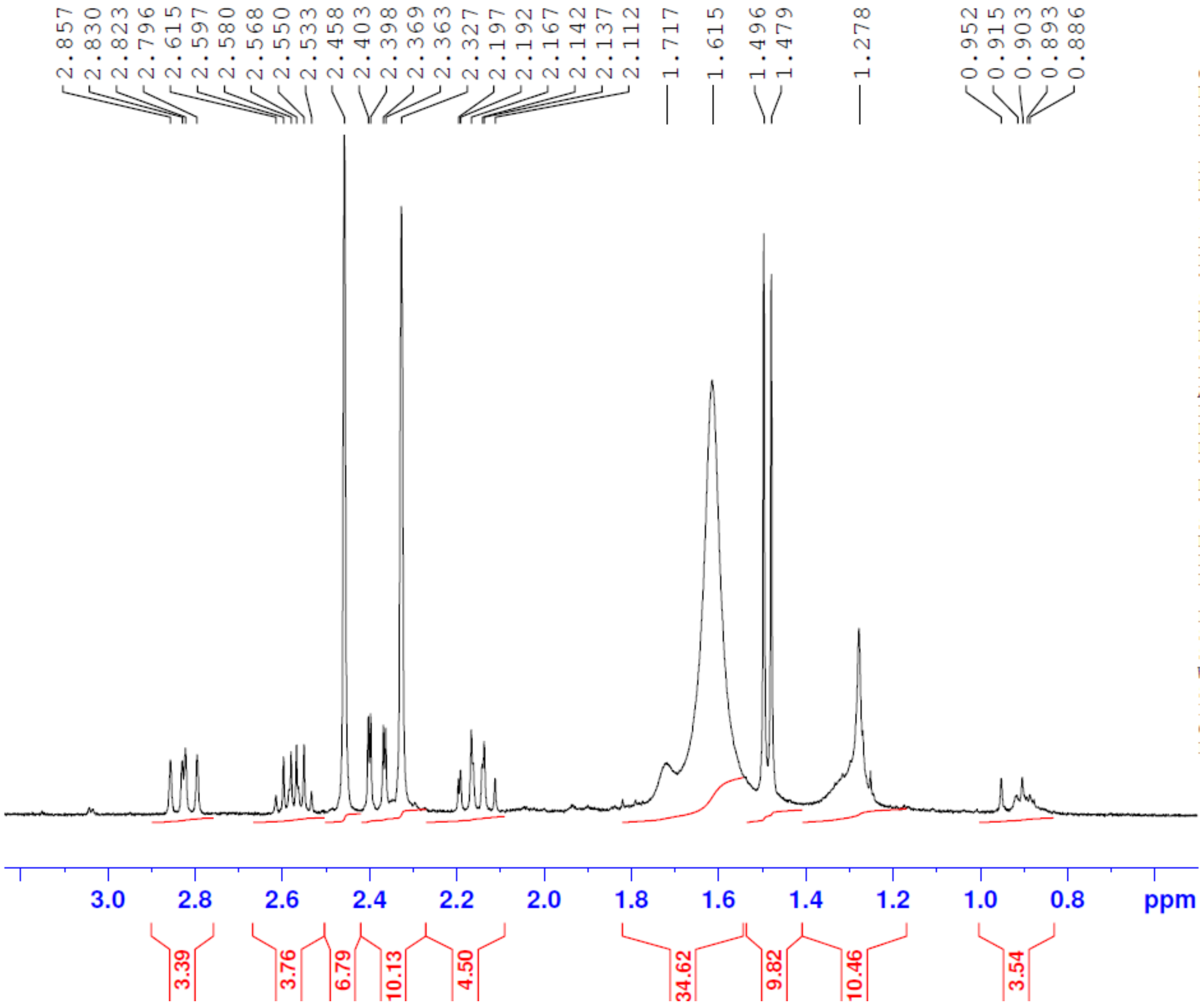

Figure S4a. ^{1}H NMR spectrum of compound **3**.

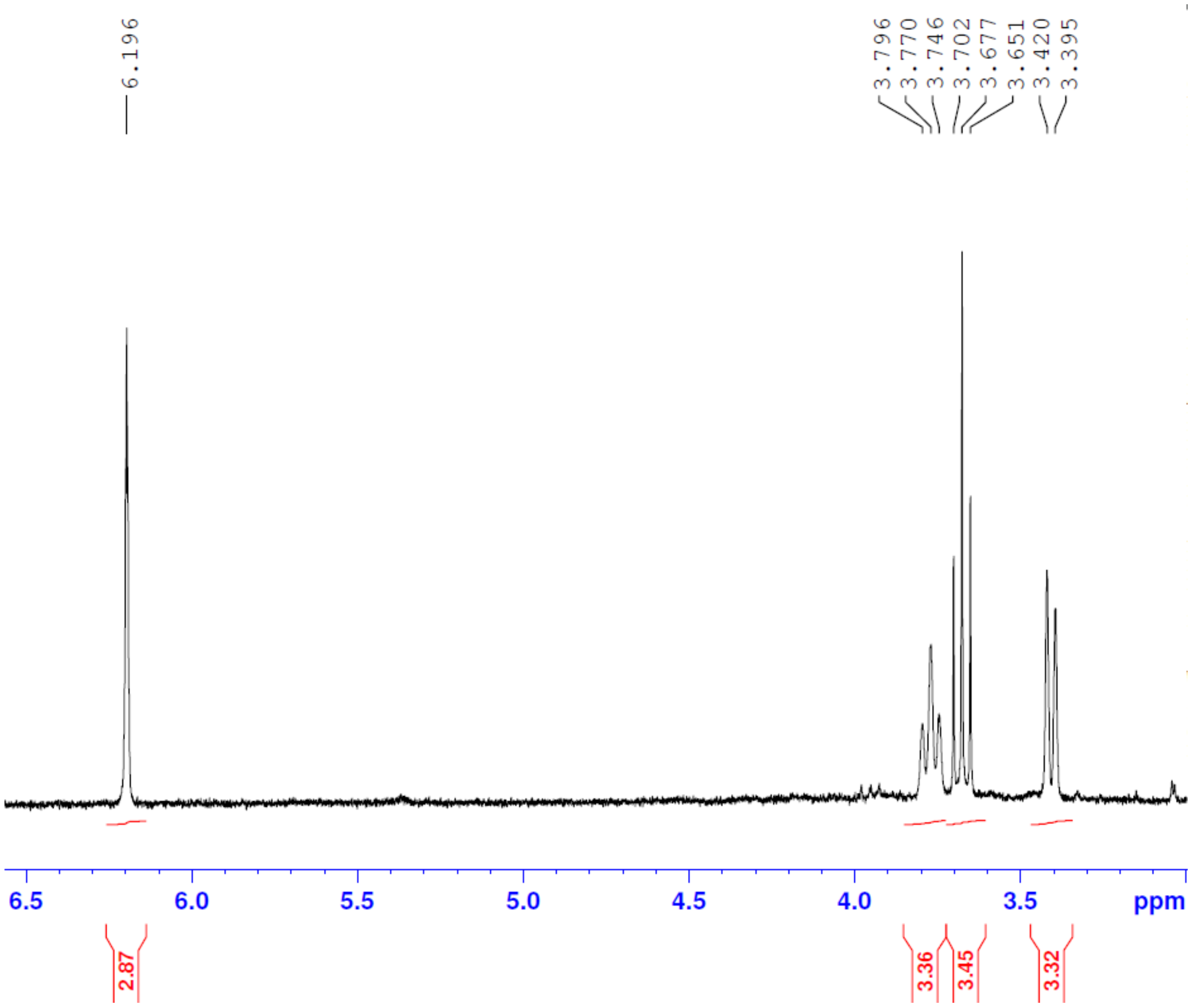

Figure S4b. ^1H NMR spectrum of compound **3**.

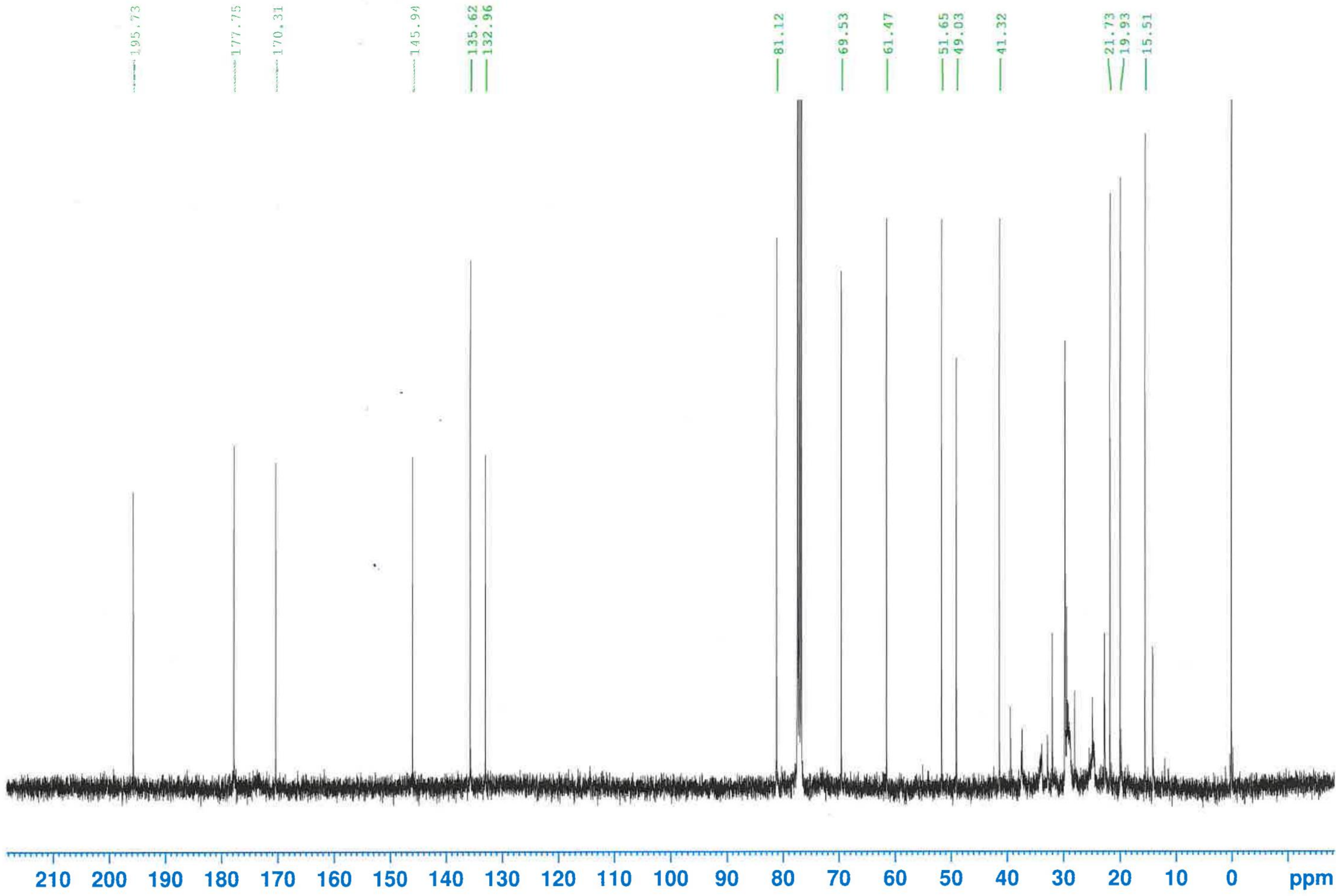

Figure S5. ^{13}C NMR spectrum of compound **3**.

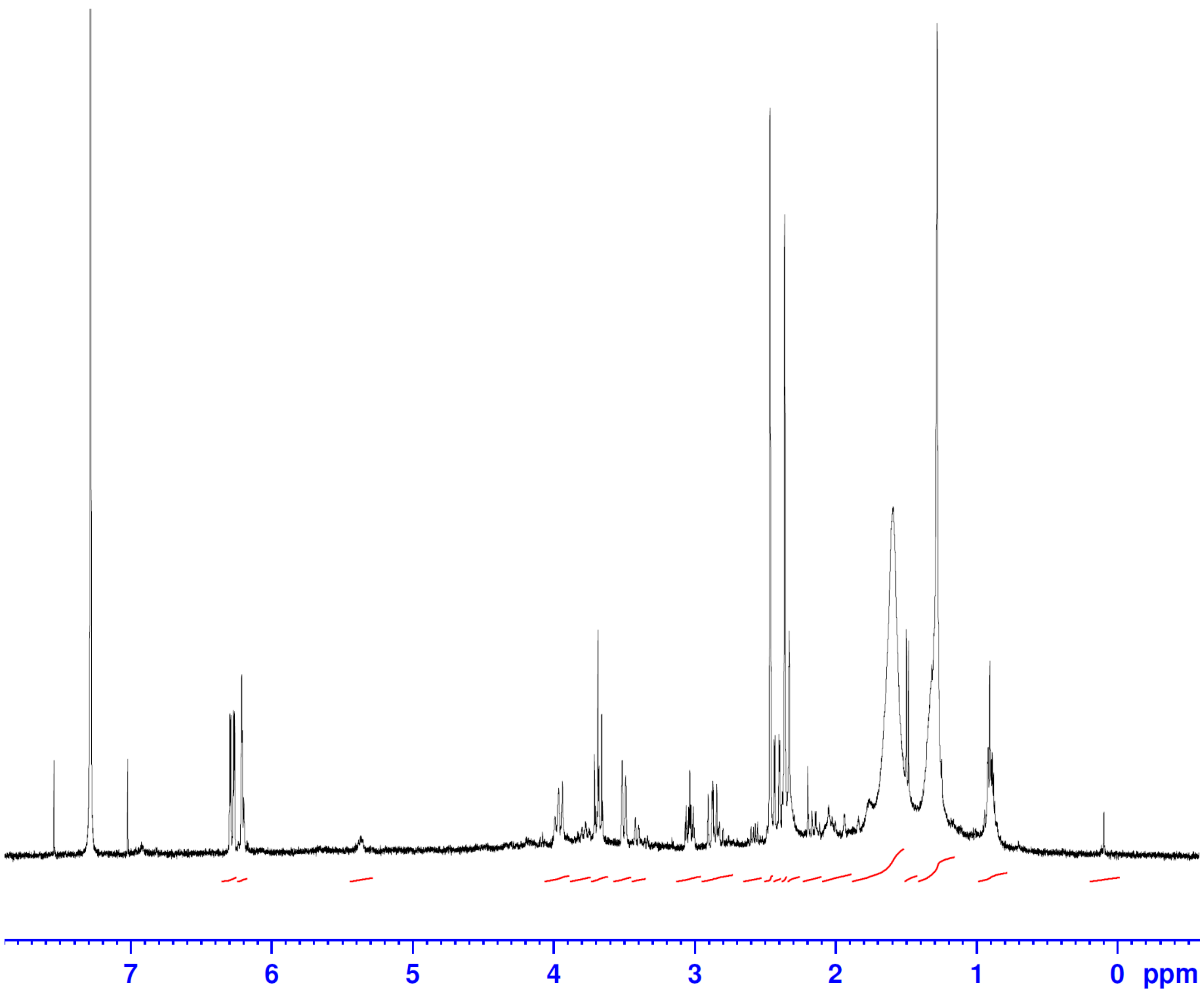

Figure S6. ^{1}H NMR spectrum of compound **4**.

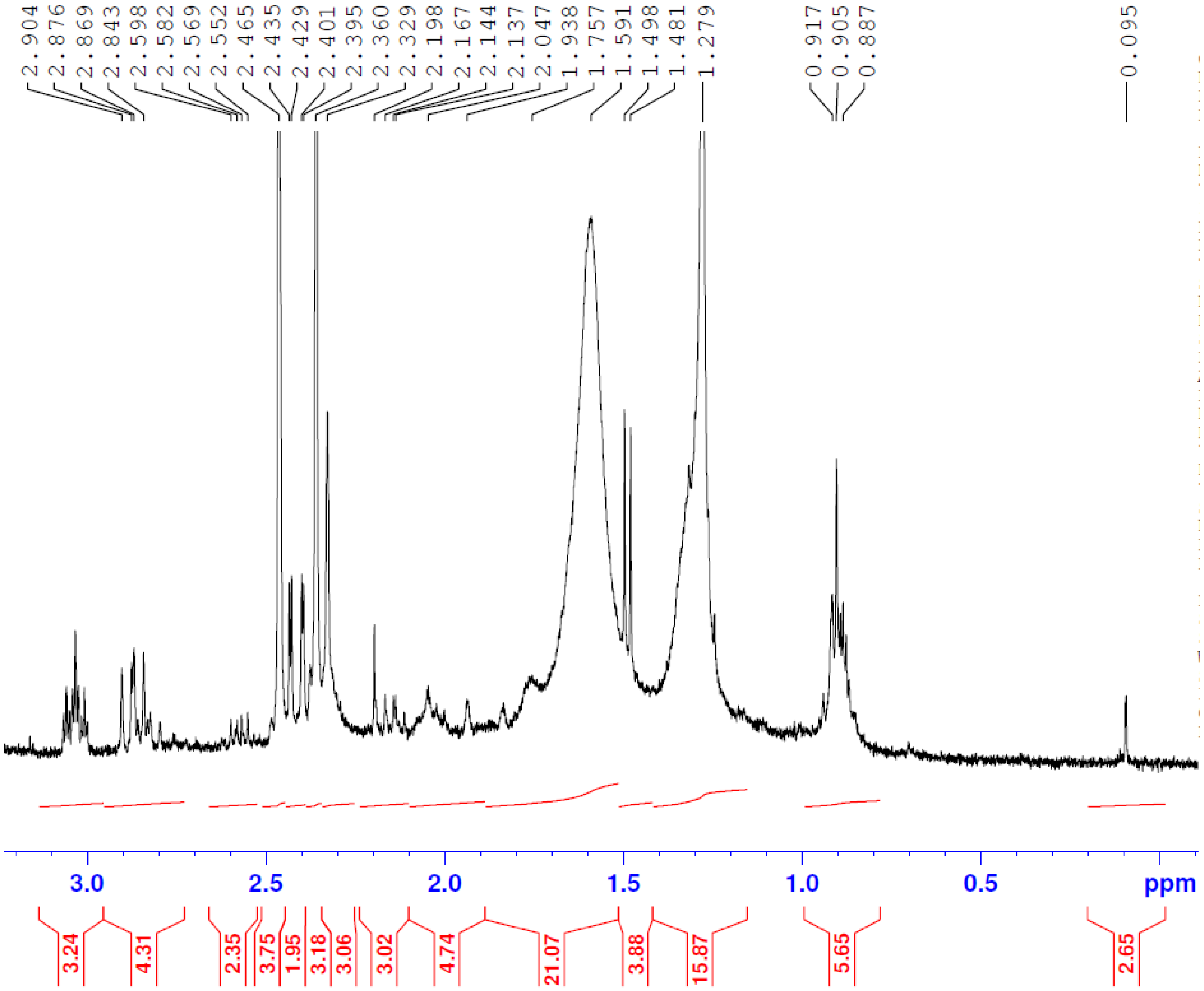

Figure S6a. ¹H NMR spectrum of compound **4**.

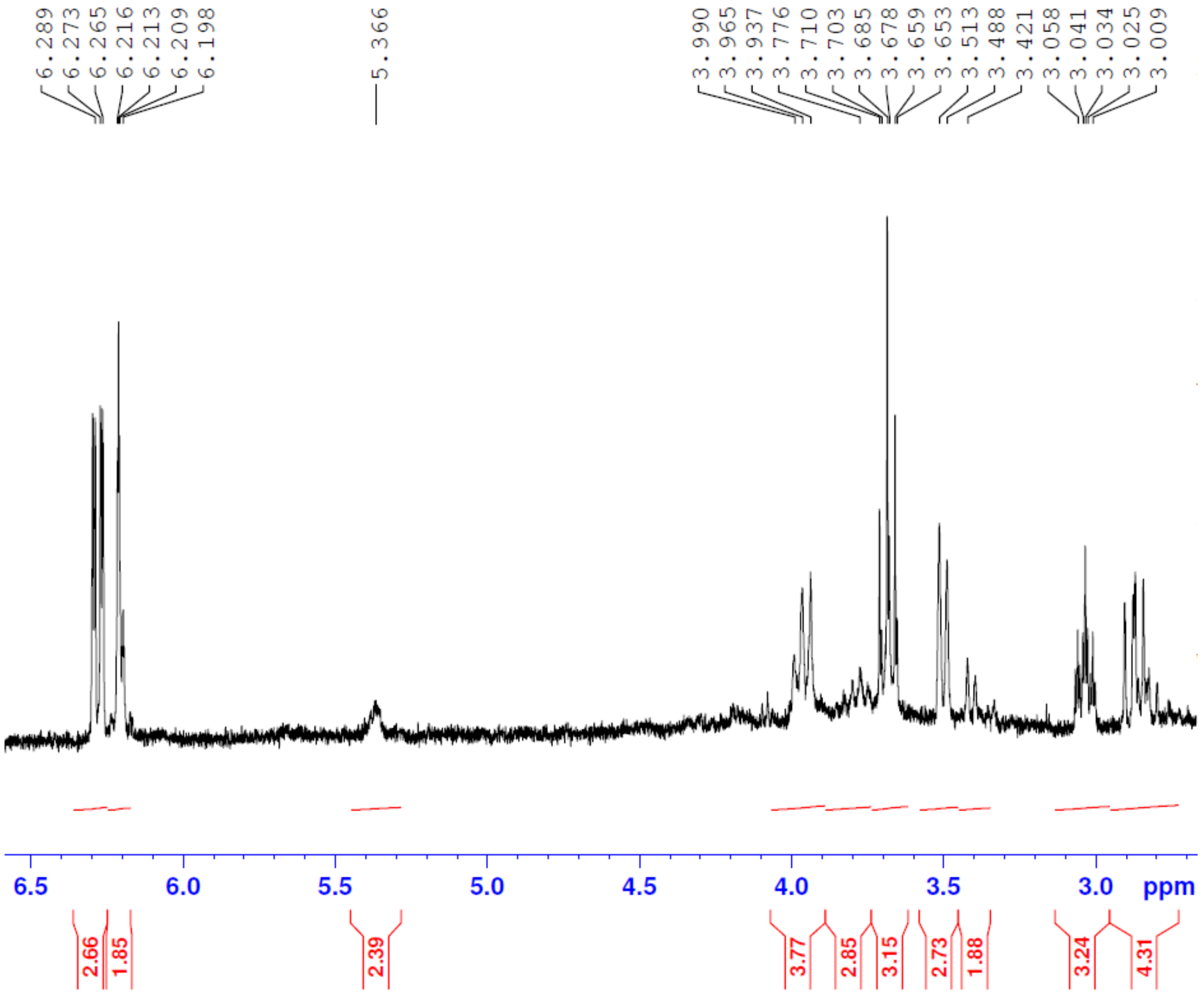

Figure S6b. ^{1}H NMR spectrum of compound **4**.

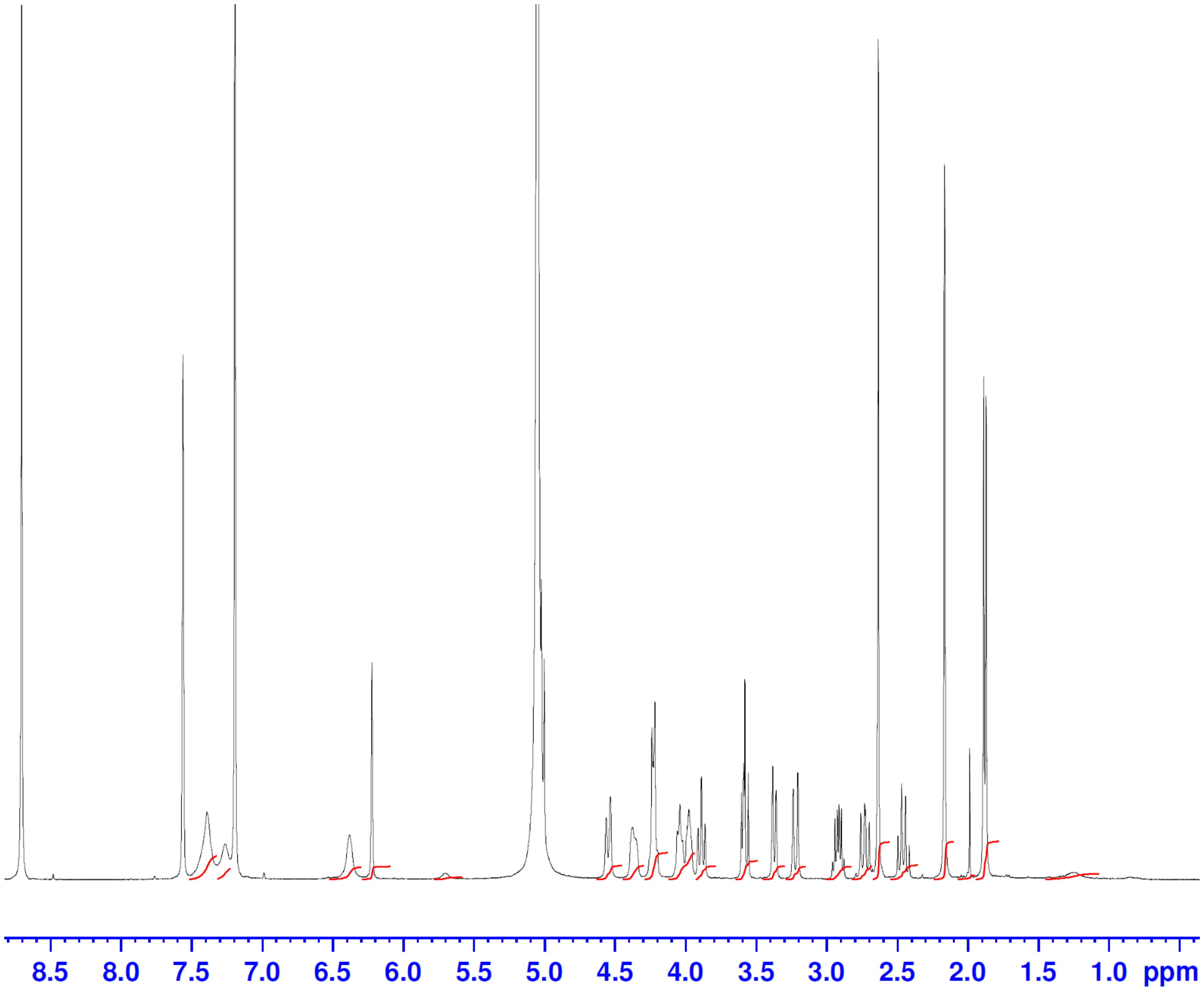

Figure S7. ^1H NMR spectrum of compound **5**.

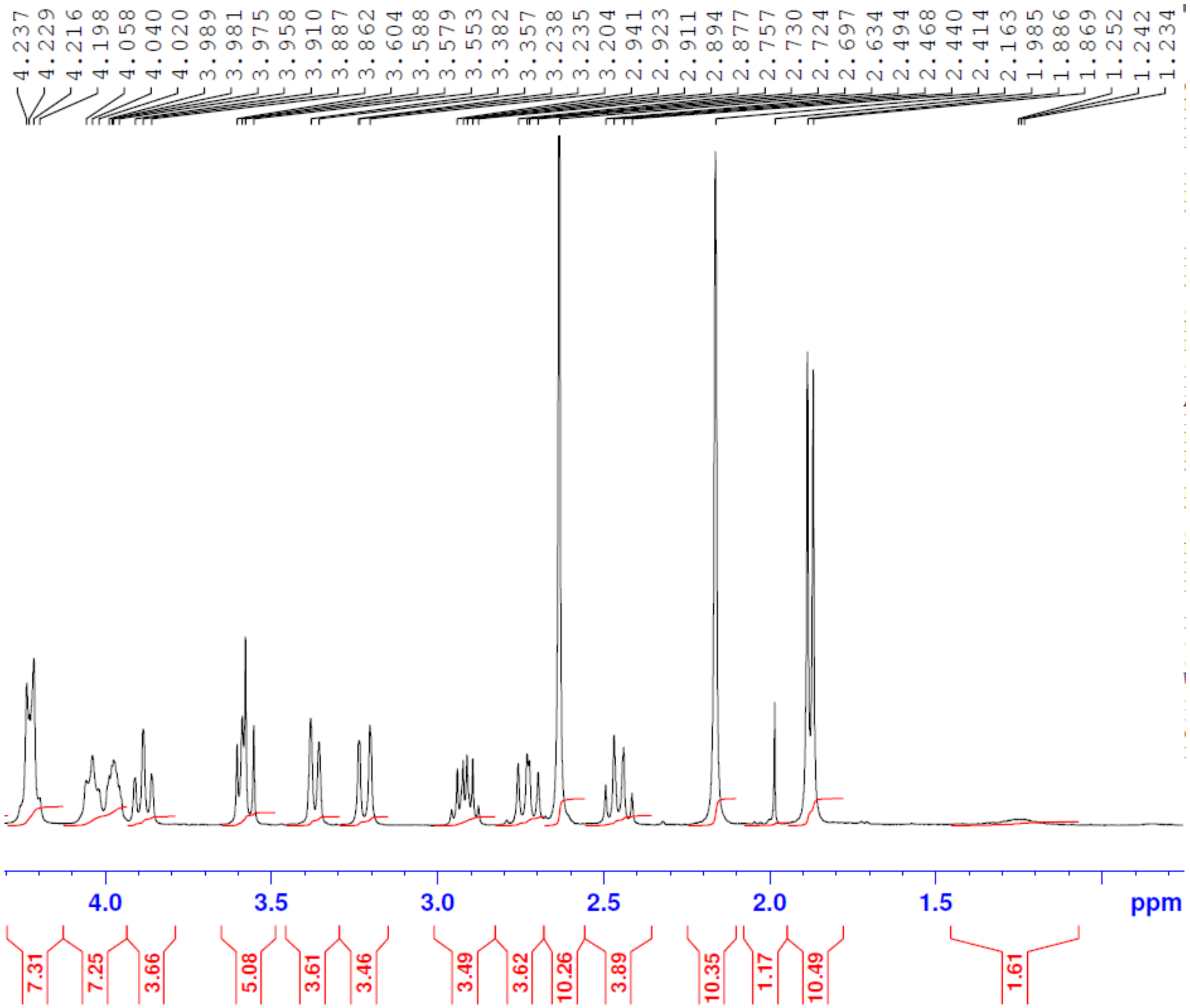

Figure S7a. ^{1}H NMR spectrum of compound **5**.

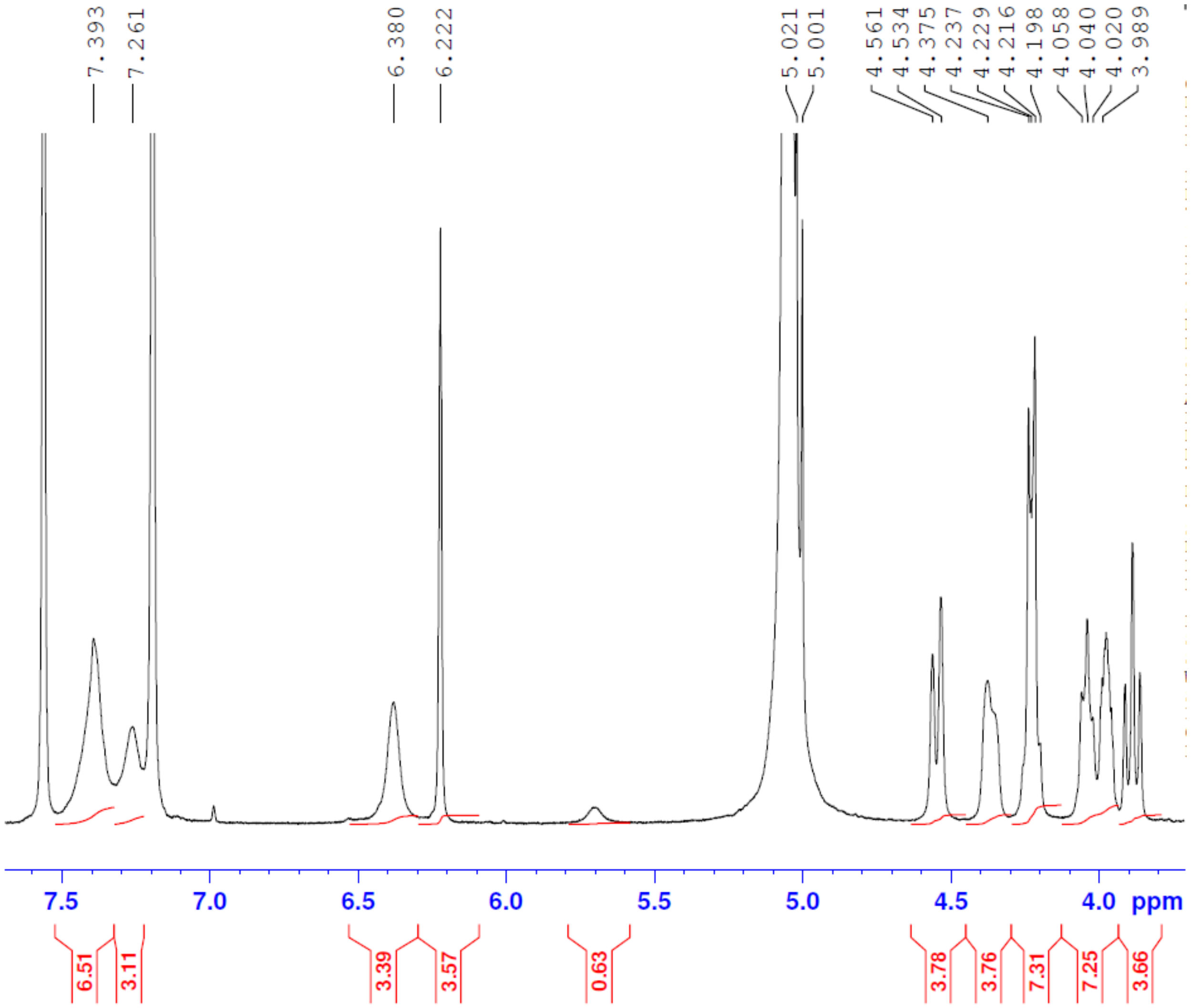

Figure S7b. ^{1}H NMR spectrum of compound **5**.

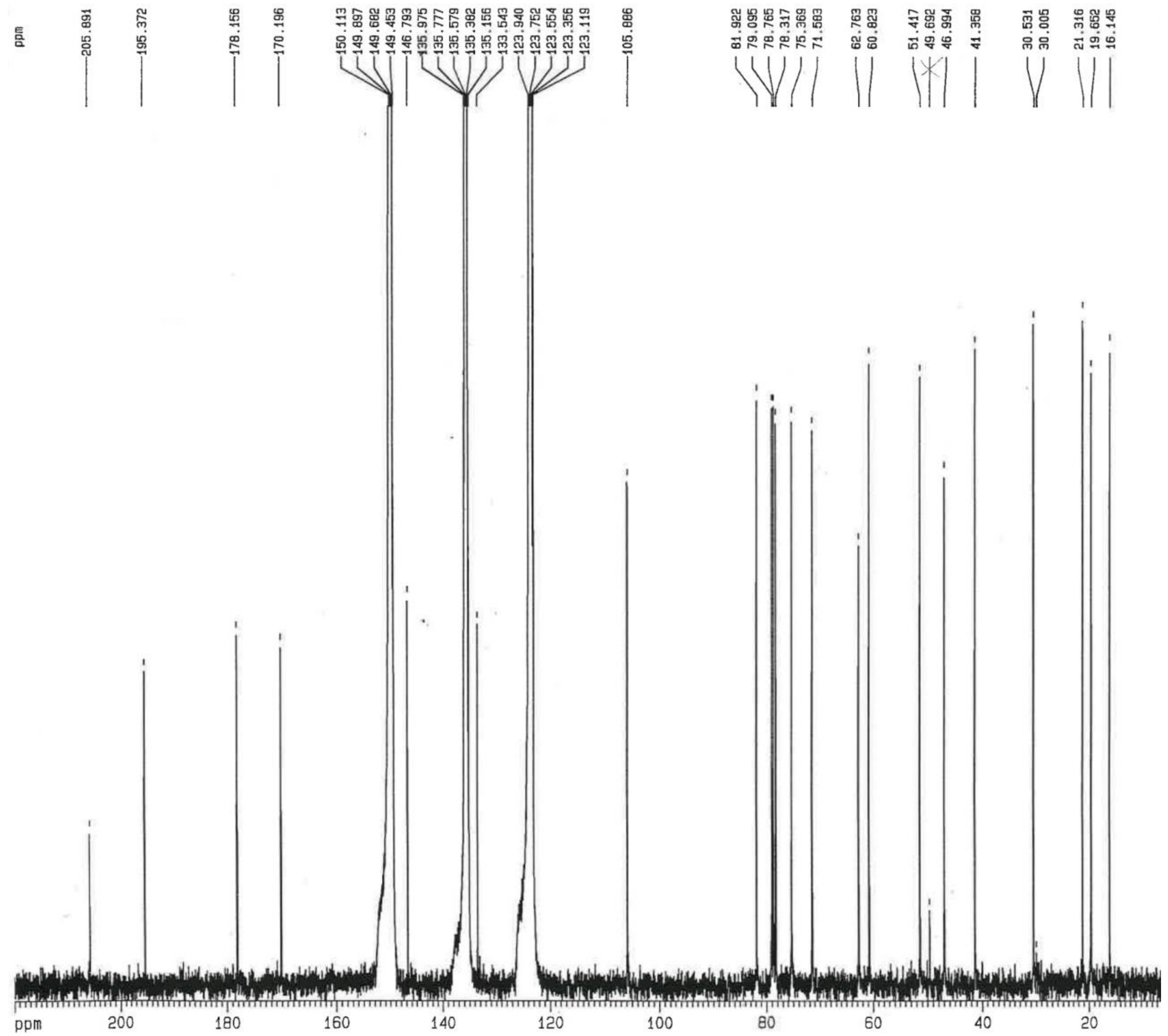

Figure S8. ^{13}C NMR spectrum of compound **5**.

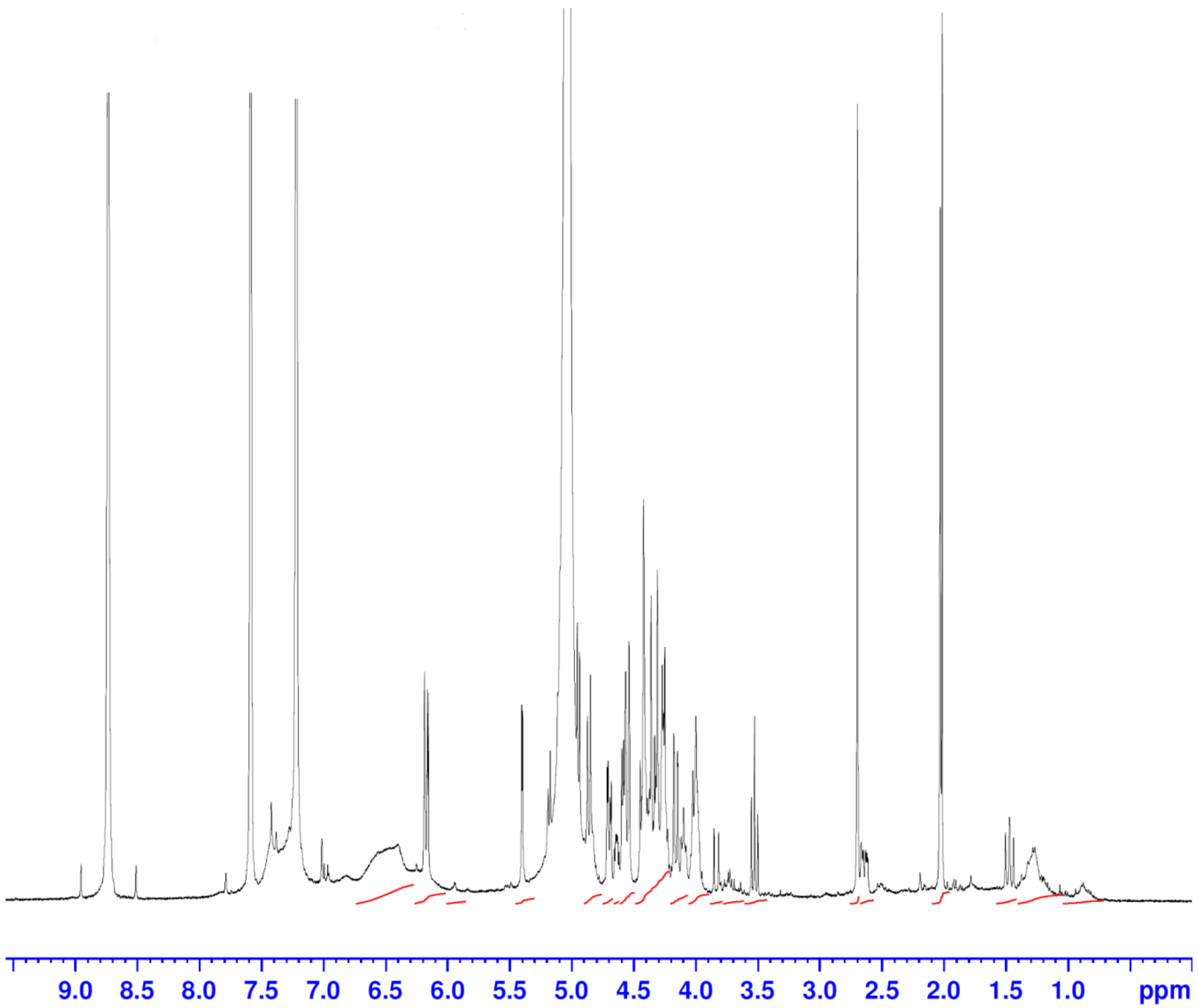

Figure S9. ^{1}H NMR spectrum of compound **6**.

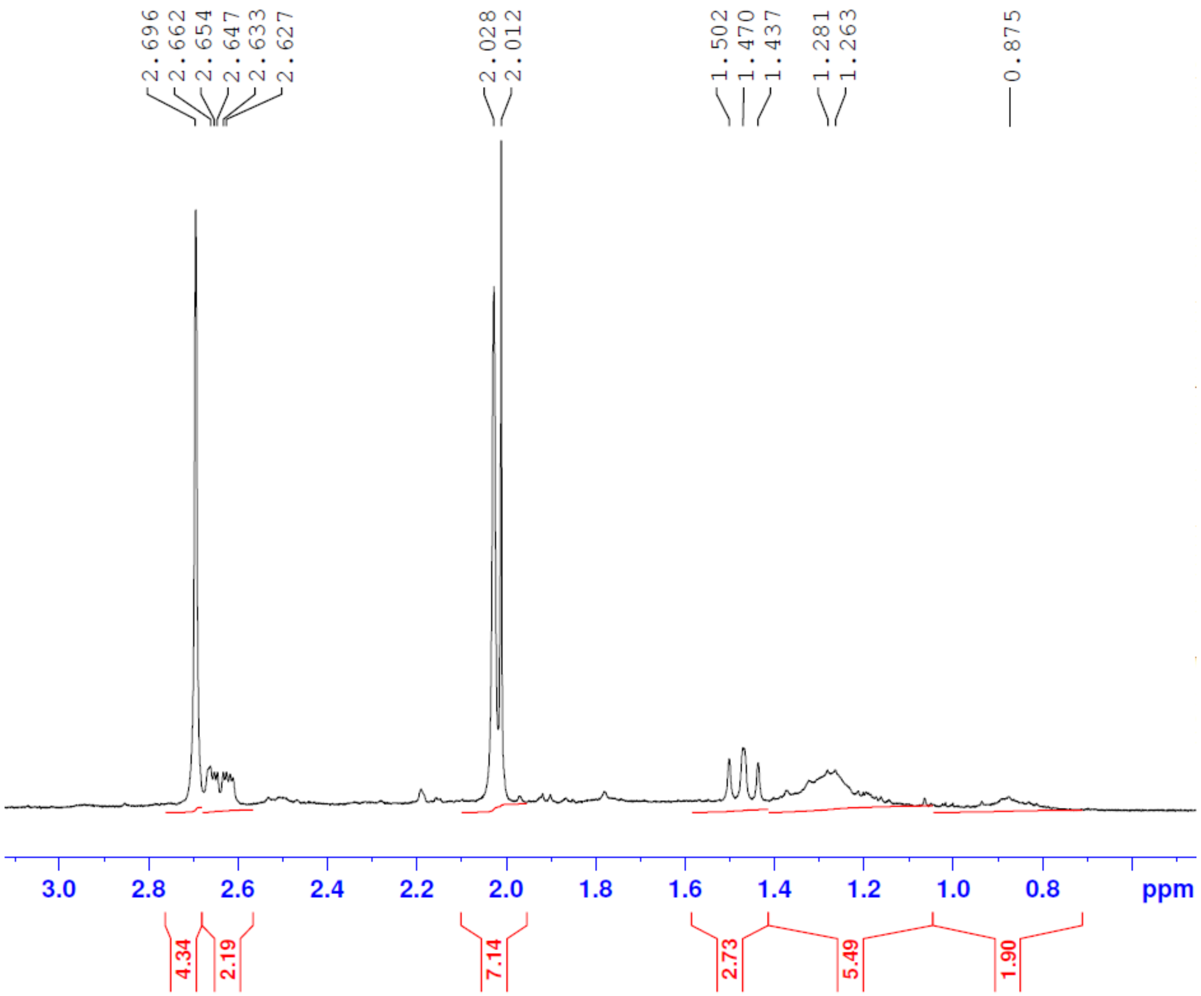

Figure S9a. ^{1}H NMR spectrum of compound **6**.

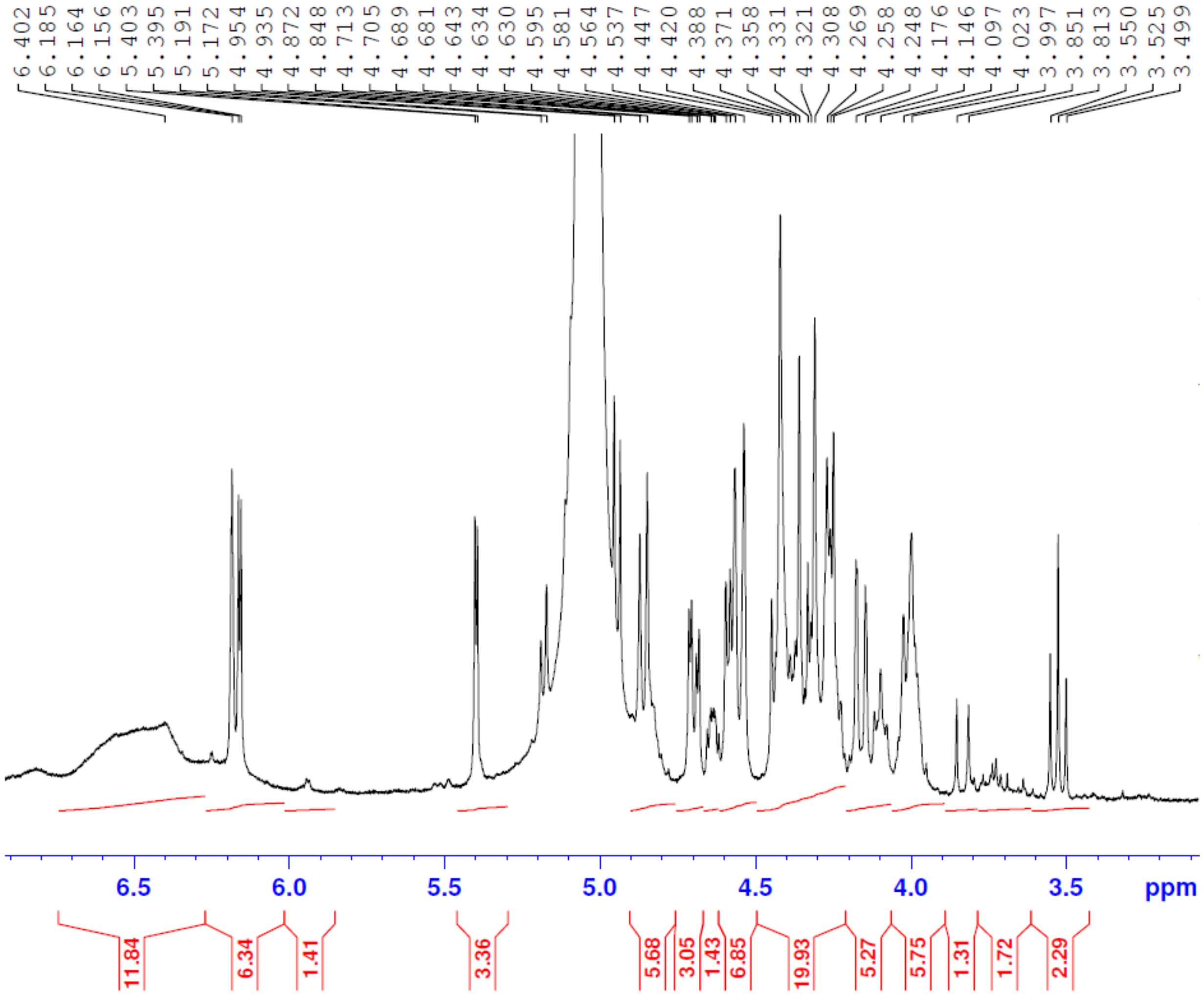

Figure S9b. ^{1}H NMR spectrum of compound **6**.

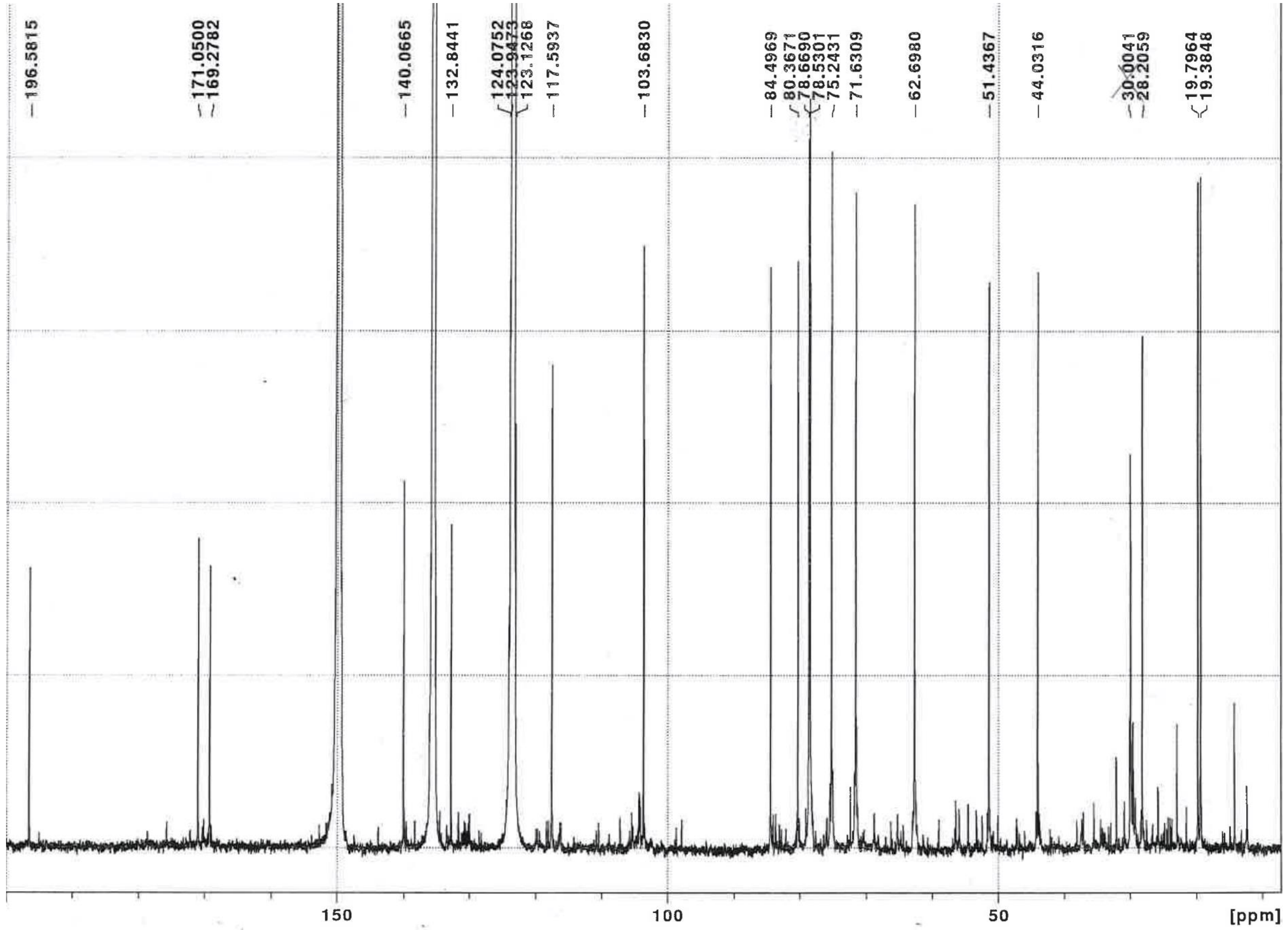

Figure S10. ^{13}C NMR spectrum of compound **6**.

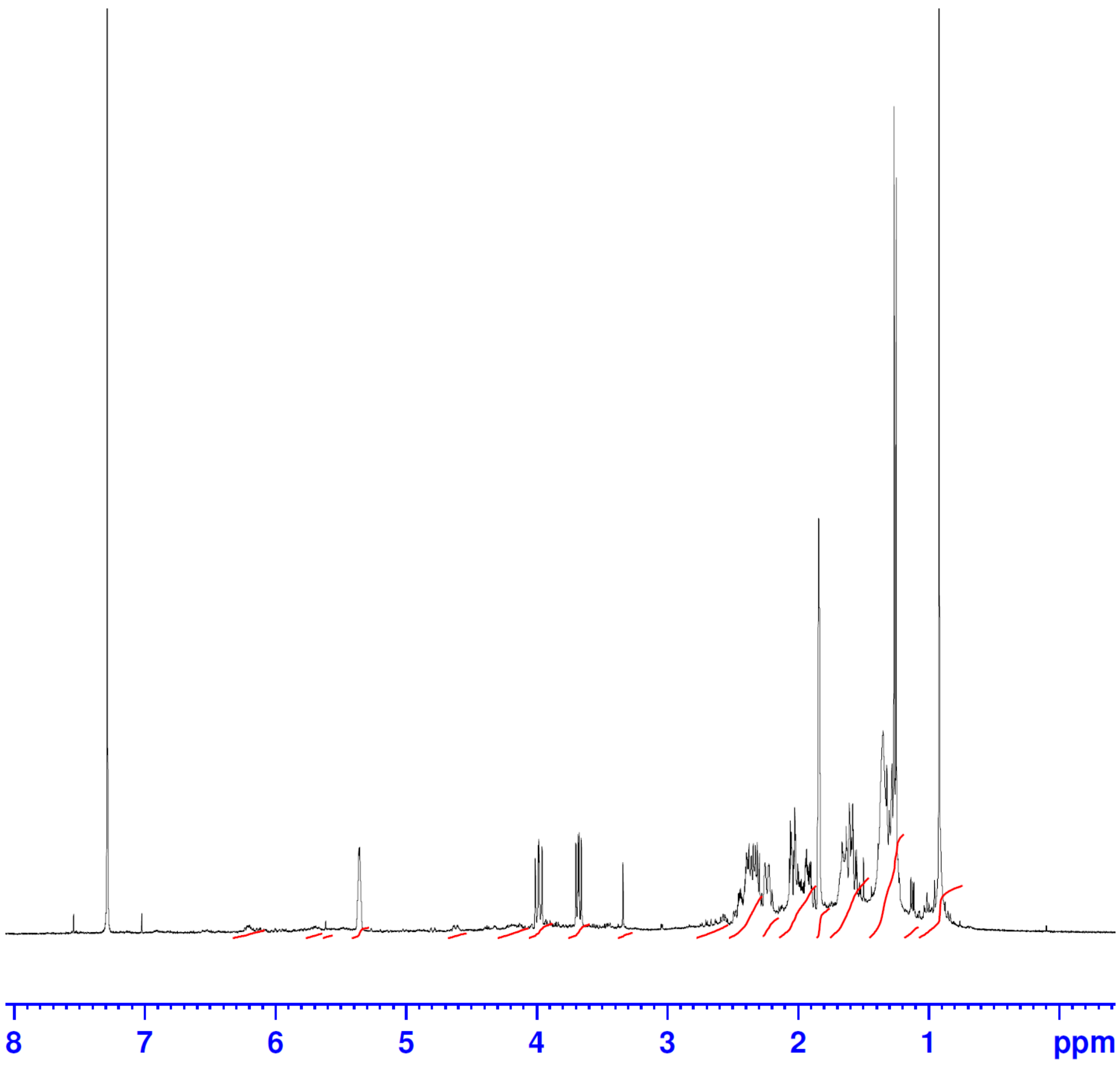

Figure S11. ^{1}H NMR spectrum of compound **7**.

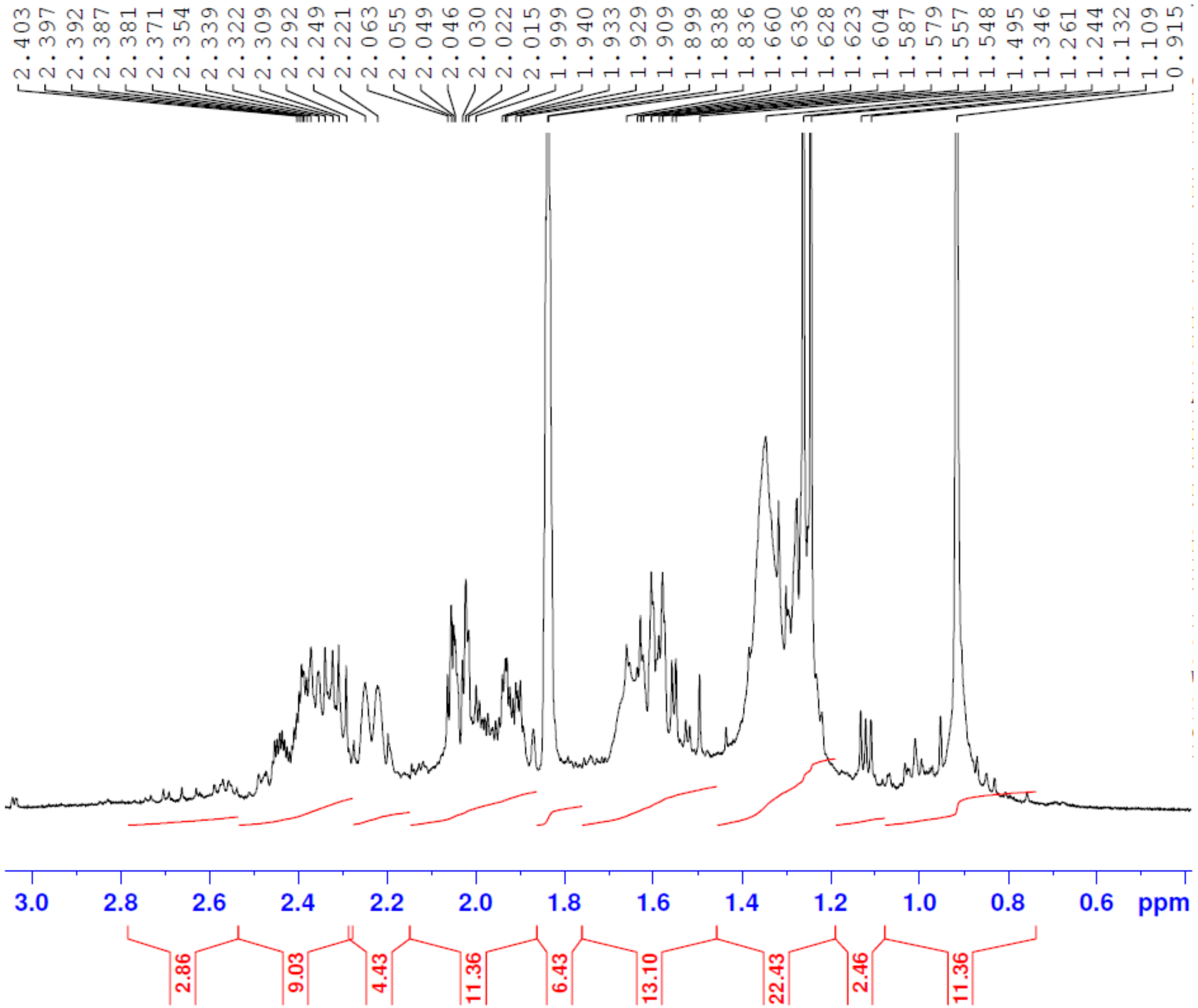

Figure S11a. ^{1}H NMR spectrum of compound **7**.

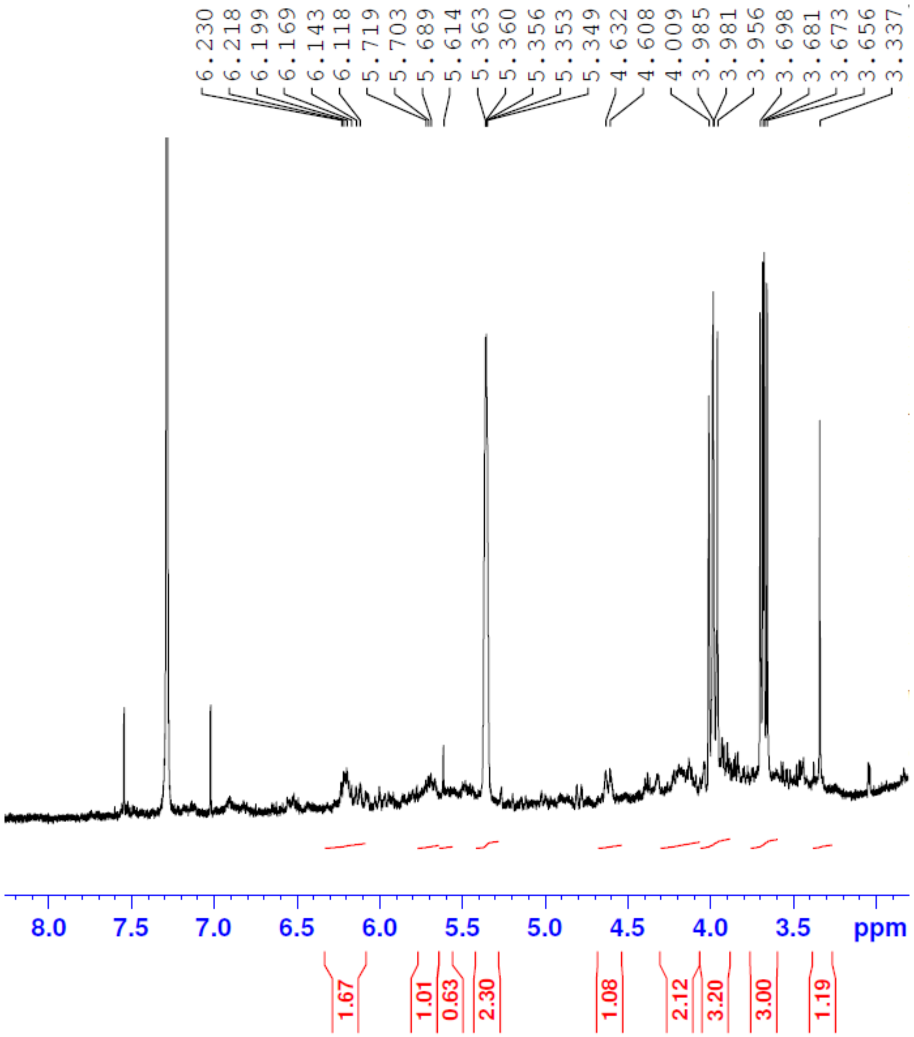

Figure S11b. ^{1}H NMR spectrum of compound **7**.

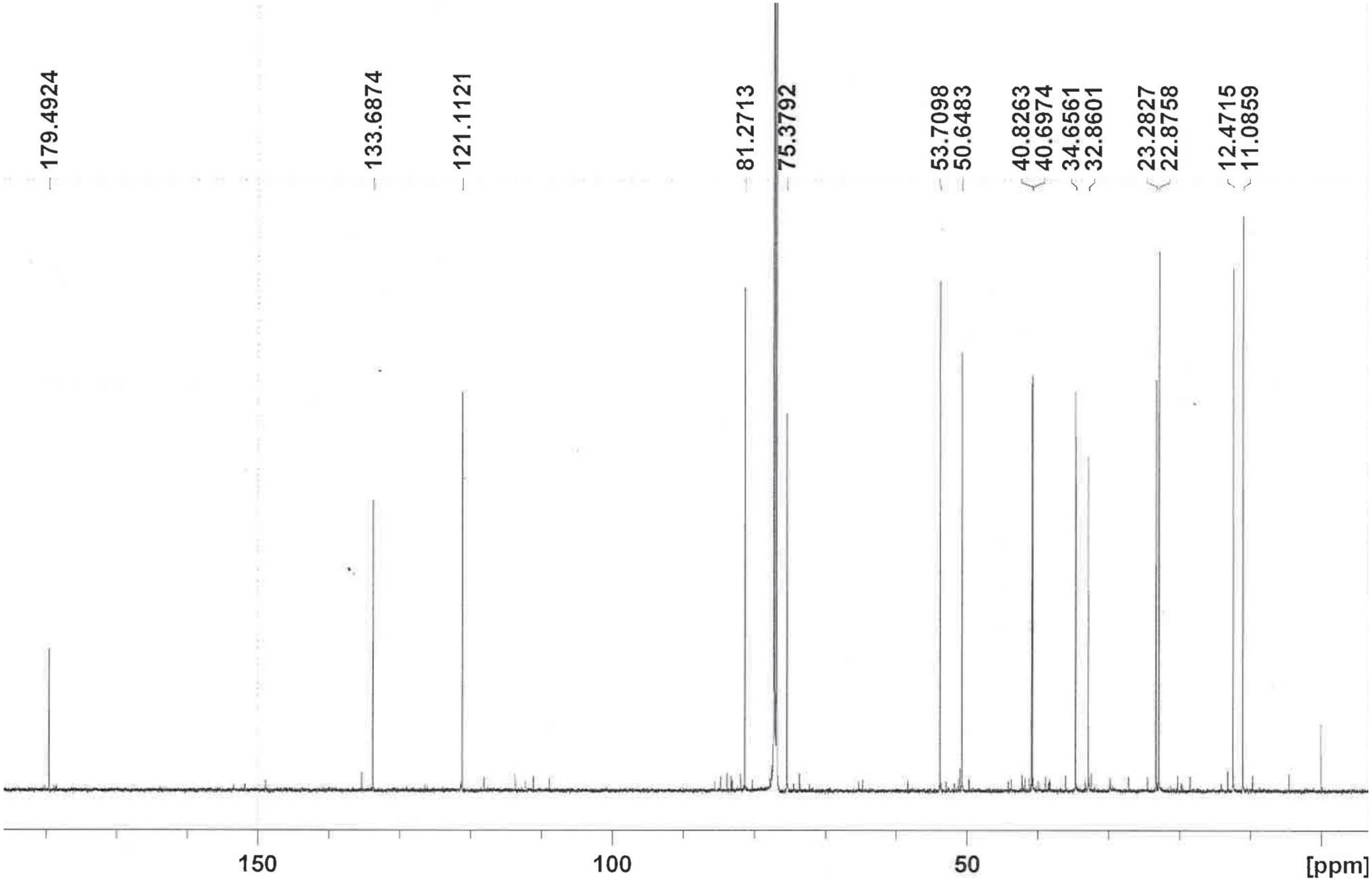

Figure S12. ^{13}C NMR spectrum of compound **7**.

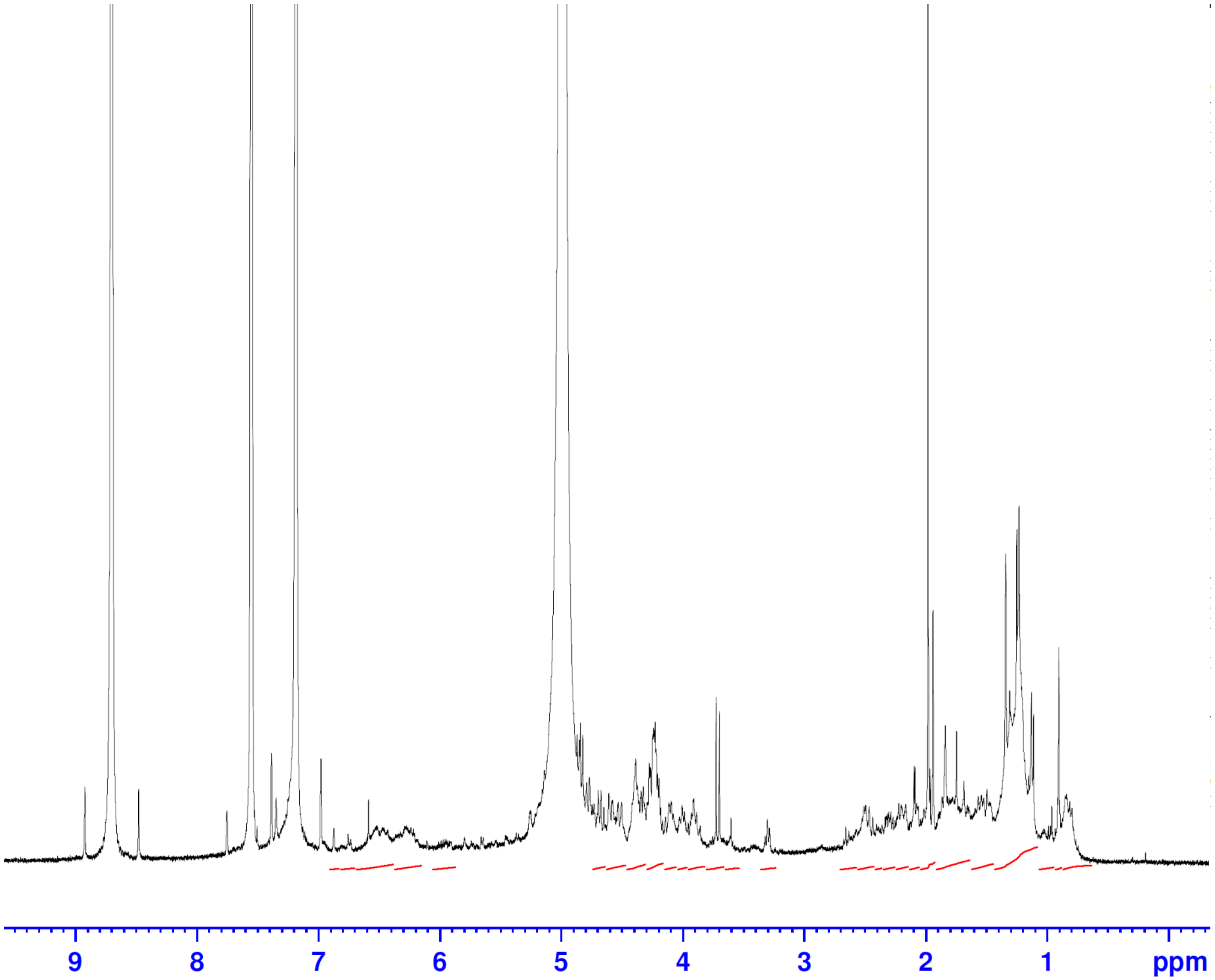

Figure S13. ^{1}H NMR spectrum of compound **8**.

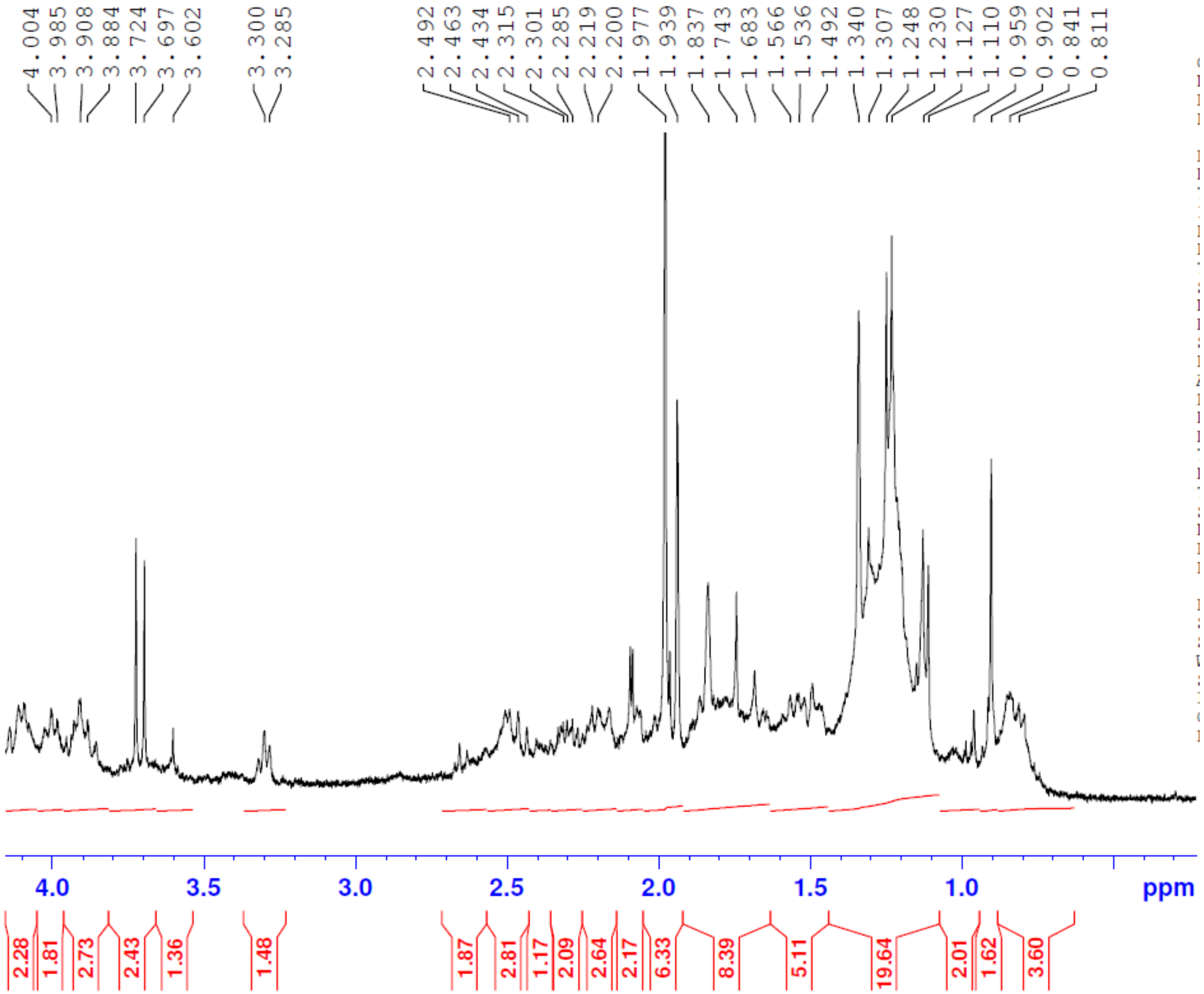

Figure S13a. ^{1}H NMR spectrum of compound **8**.

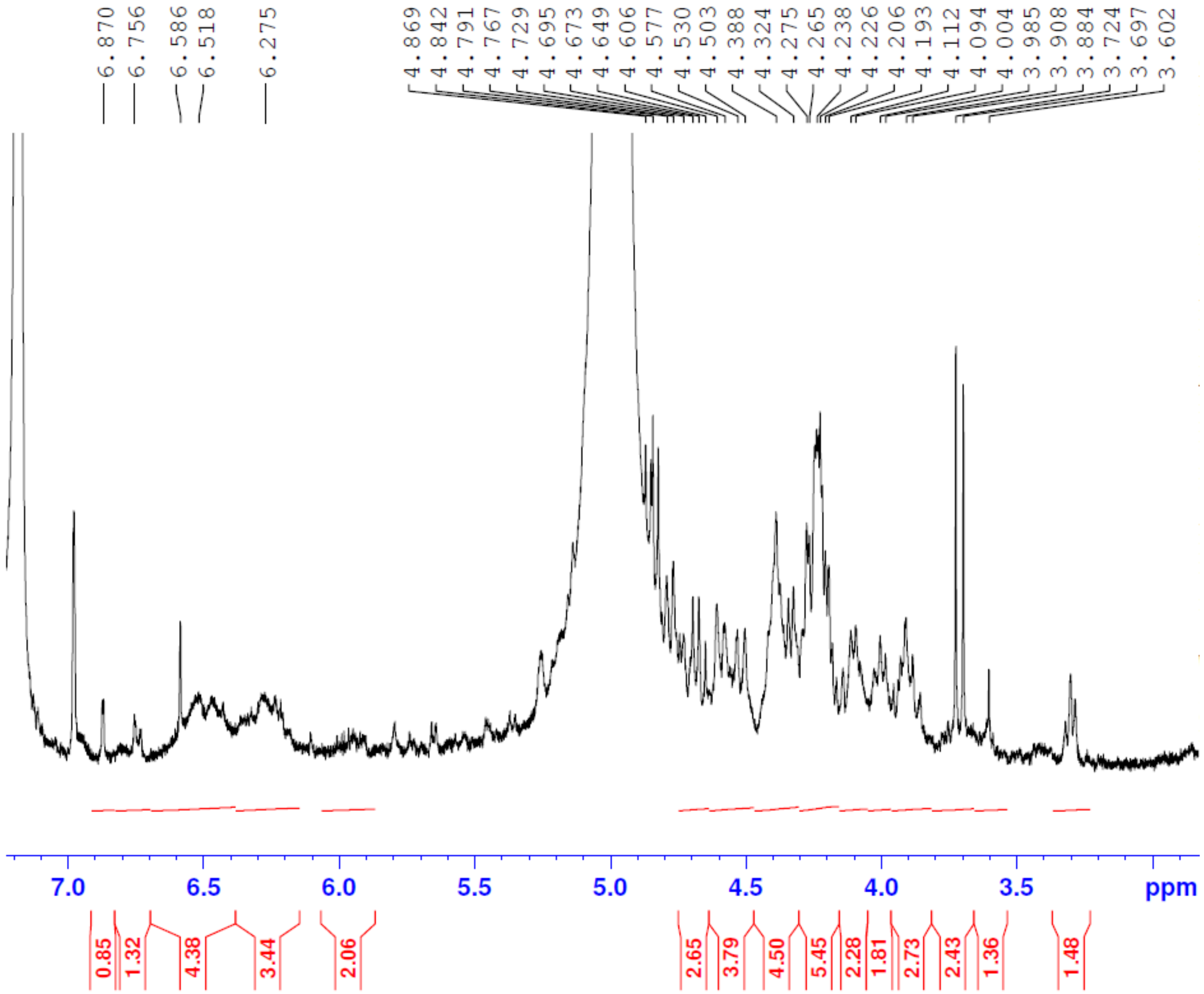

Figure S13b. ^{1}H NMR spectrum of compound **8**.

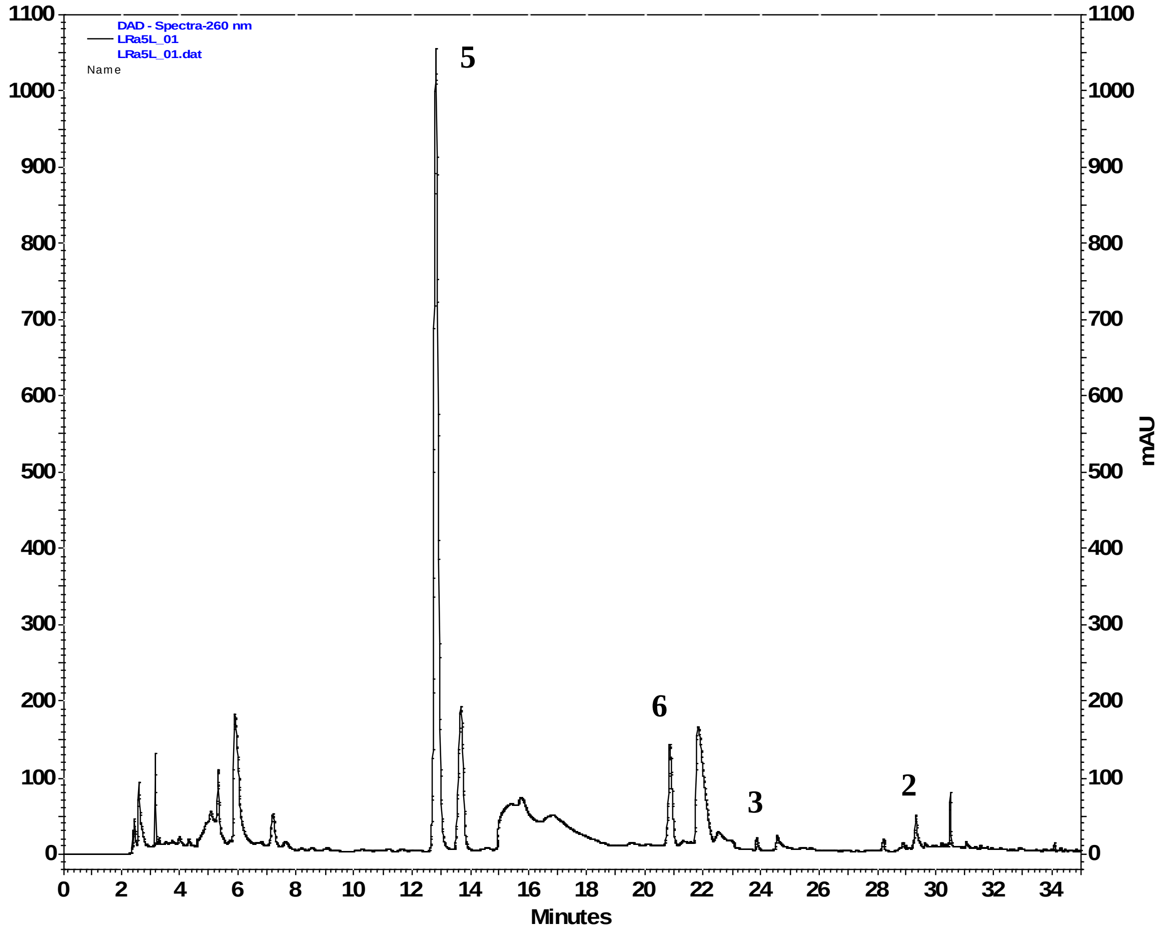

Figure S14. HPLC/PAD chromatogram of a methanol extract from roots of *Lactuca racemosa*, acquired at 260 nm: **5** – deacetylmatricarin 8-β-glucopyranoside; **6** – 11,13-dehydrolactuside C; **3** – deacetylmatricarin; **2** – matricarin.
